# Supplementary material for: Identification and characterization of calcium binding protein, spermatid-associated 1 (CABS1)# in selected human tissues and fluids
Source: PLoS One. 2024 May 16;19(5):e0301855. doi: 10.1371/journal.pone.0301855 (PMC11098423; doi:10.1371/journal.pone.0301855)
Supplement: S1 Fig — (PDF) [file pone.0301855.s001.pdf]

Supplemental Figure 1: Binding of Monoclonal antibody 4D1 to peptide 3 (AA40 to 60) of CABS1

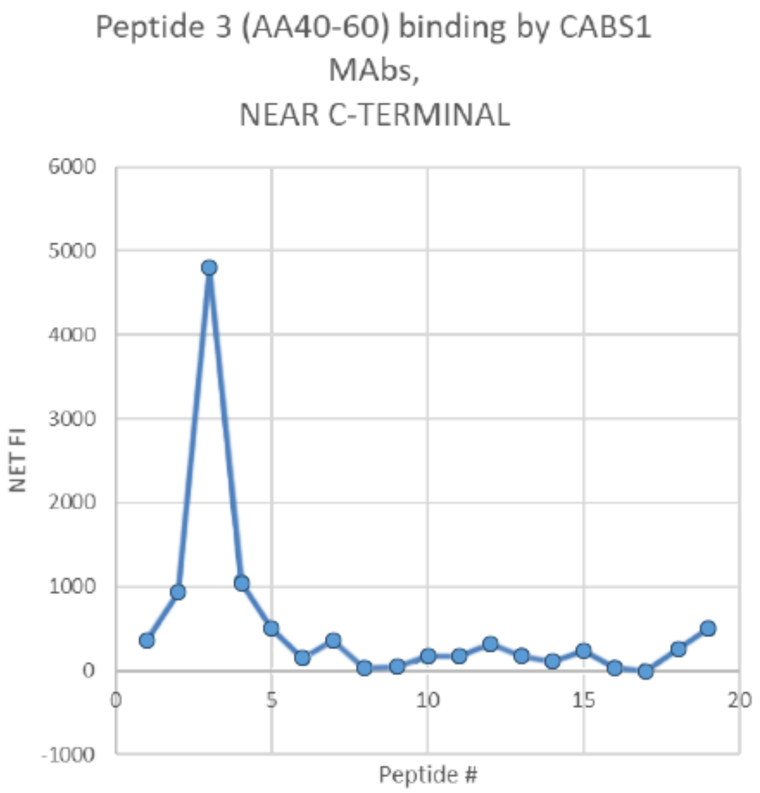

Peptide#	4D1
1	359
2	929
3	4795
4	1065
5	494
6	150
7	381
8	30
9	6
10	158
11	170
12	308
13	200
14	109
15	242
16	30
17	-1
18	254
19	508

I

This Elisa immunoassay involves the mAb 4D1 bound to a goat anti-mouse antibody Fc fragment on the plate surface. It then was exposed to the CABS1 biotin-peptide library and following extensive washing of unbound peptide-biotin complexes, the specific bound peptide-biotin complex was detected by SA-Fluorescein.
