# Supplementary figures and images for: Identification and characterization of calcium binding protein, spermatid-associated 1 (CABS1)# in selected human tissues and fluids
Source: PLoS One. 2024 May 16;19(5):e0301855. doi: 10.1371/journal.pone.0301855 (PMC11098423; doi:10.1371/journal.pone.0301855)

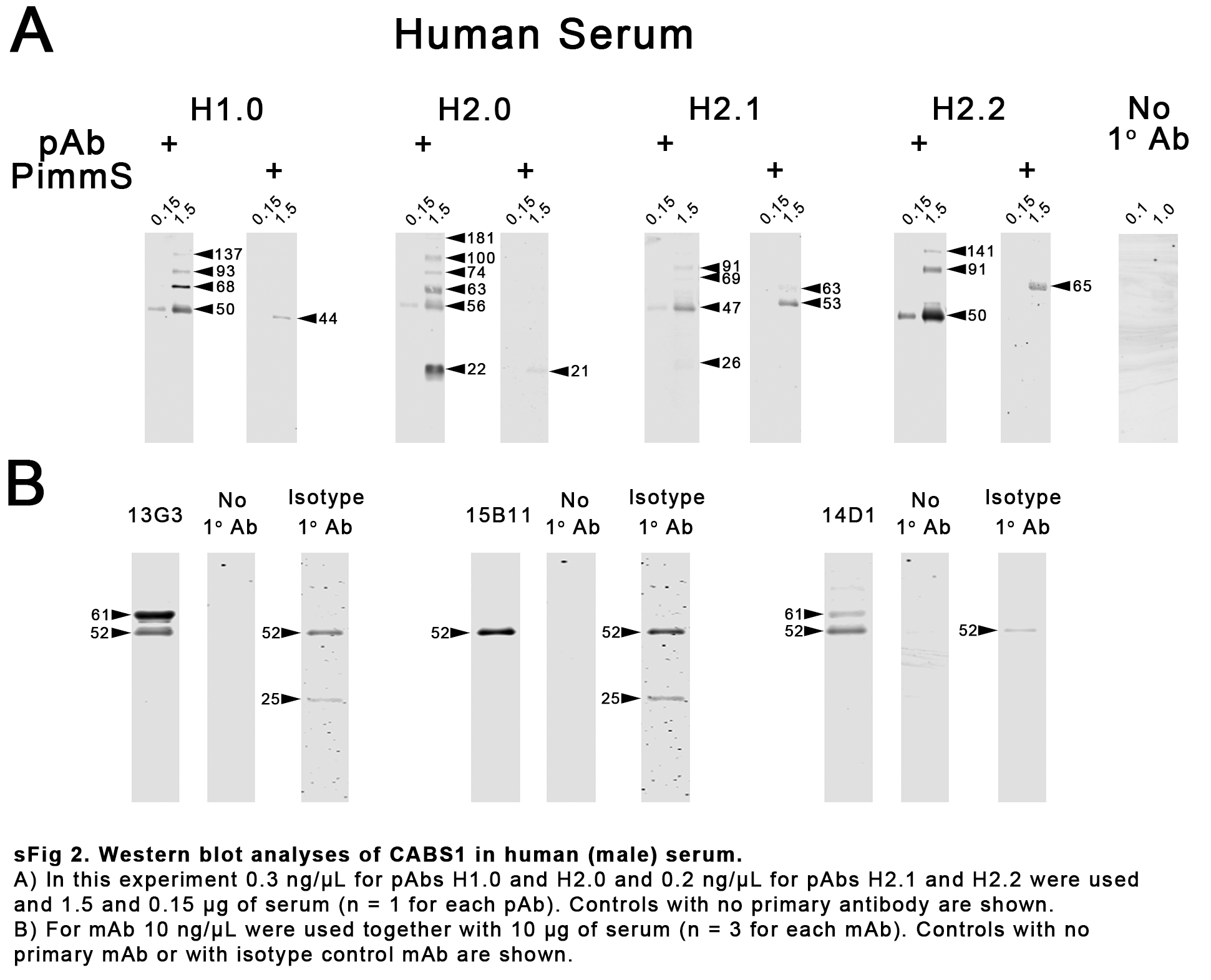

Supplement: S2 Fig — (TIF) [file pone.0301855.s002.tif]
