# Supplementary material for: Identification and characterization of calcium binding protein, spermatid-associated 1 (CABS1)# in selected human tissues and fluids
Source: PLoS One. 2024 May 16;19(5):e0301855. doi: 10.1371/journal.pone.0301855 (PMC11098423; doi:10.1371/journal.pone.0301855)
Supplement: S3 Fig — (PDF) [file pone.0301855.s003.pdf]

Legends of supplemental immunofluorescence images of submandibular glands

S3.1 Fig. Intralobular salivary collecting duct and adjacent serous acini stained with anti-CABS1 pAb H1.0. Green = CABS1, Red = autofluorescence (RBCs etc.), Blue = nuclei (DAPI).

S3.2 Fig. Mucinous and serous acinar cells are stained for CABS1 (pAb H1.0). Green = CABS1, Red = autofluorescence (RBCs etc.), Blue = nuclei (DAPI).

S3.3 Fig. CABS1 H2.0 pAb shows staining of selected abluminal epithelial cells in the salivary collecting and excretory ducts (yellow arrows). Insert shows preimmune serum, negative control. Green = CABS1, Red = autofluorescence, Blue = nuclei (DAPI).

S3.4 Fig. CABS1 H2.1 pAb staining of the cytoplasm of all salivary collecting duct (SD) cells and acini. Insert shows preimmune serum, negative control. Green = CABS1, Red = autofluorescence (RBCs), Blue = nuclei (DAPI).

S3.5 Fig. CABS1 H2.1 pAb staining of the cytoplasm of all epithelial cells in excretory ducts. Green = CABS1, Red = autofluorescence, Blue = nuclei (DAPI).

S3.6 Fig. Endothelial cells (green arrow) are CABS1 positive (pAb H2.1), left panel; right panel, no primary H2.1, negative control. Green = CABS1, Red = autofluorescence, Blue = nuclei (DAPI).

S3.7 Fig. CABS1 pAb H2.2 staining of specific abluminal epithelial cells of salivary collecting ducts. Insert shows preimmune serum, negative control. Green = CABS1, Red = autofluorescence (RBCs, etc.), Blue = nuclei (DAPI).

S3.8 Fig. CABS1 H2.2 antibody stains the cytoplasm of abluminal epithelial cells of excretory ducts. Green = CABS1, Red = autofluorescence (RBCs, etc.), Blue = nuclei (DAPI).

sFig 3.1

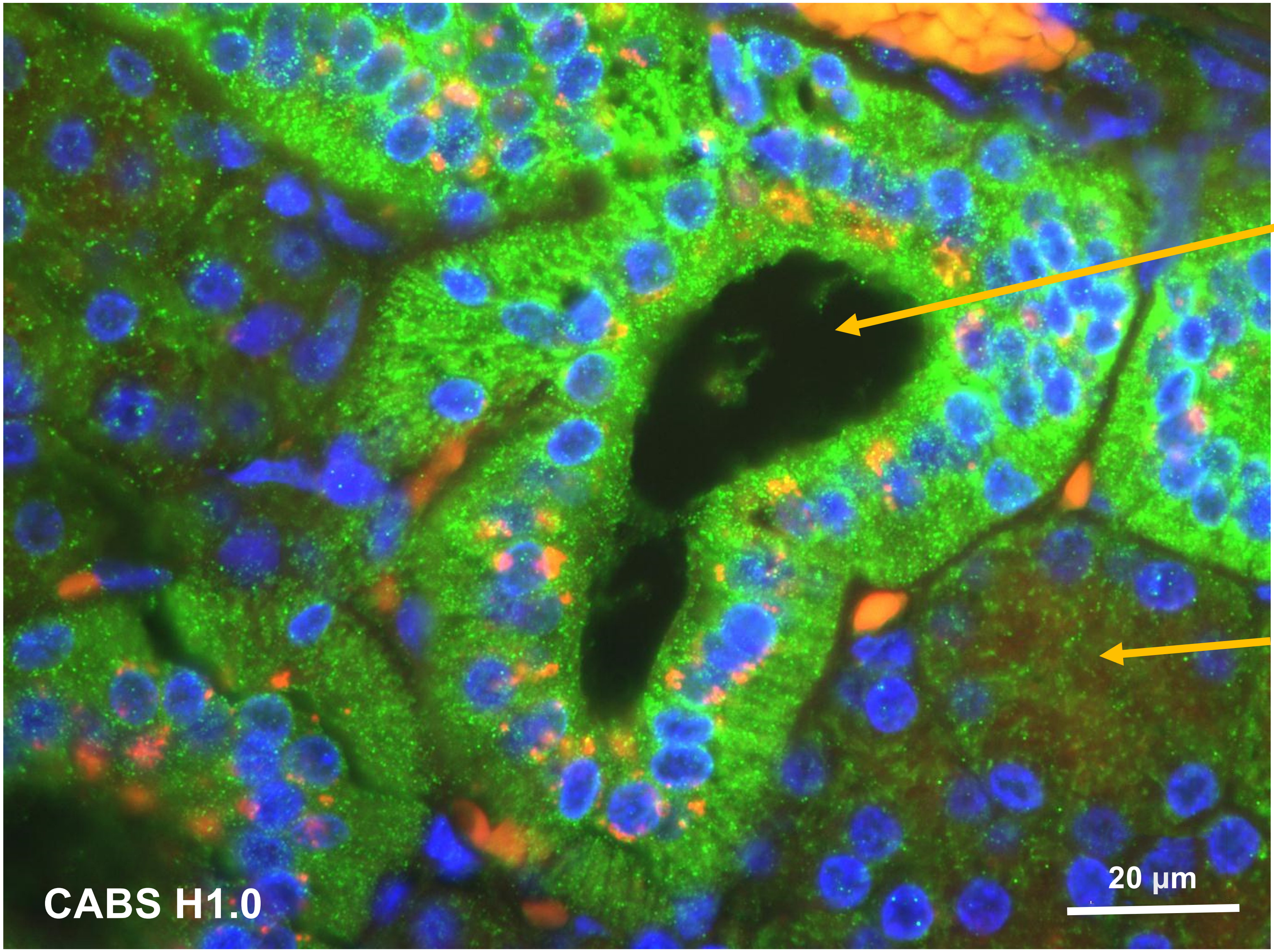

sFig 3.2

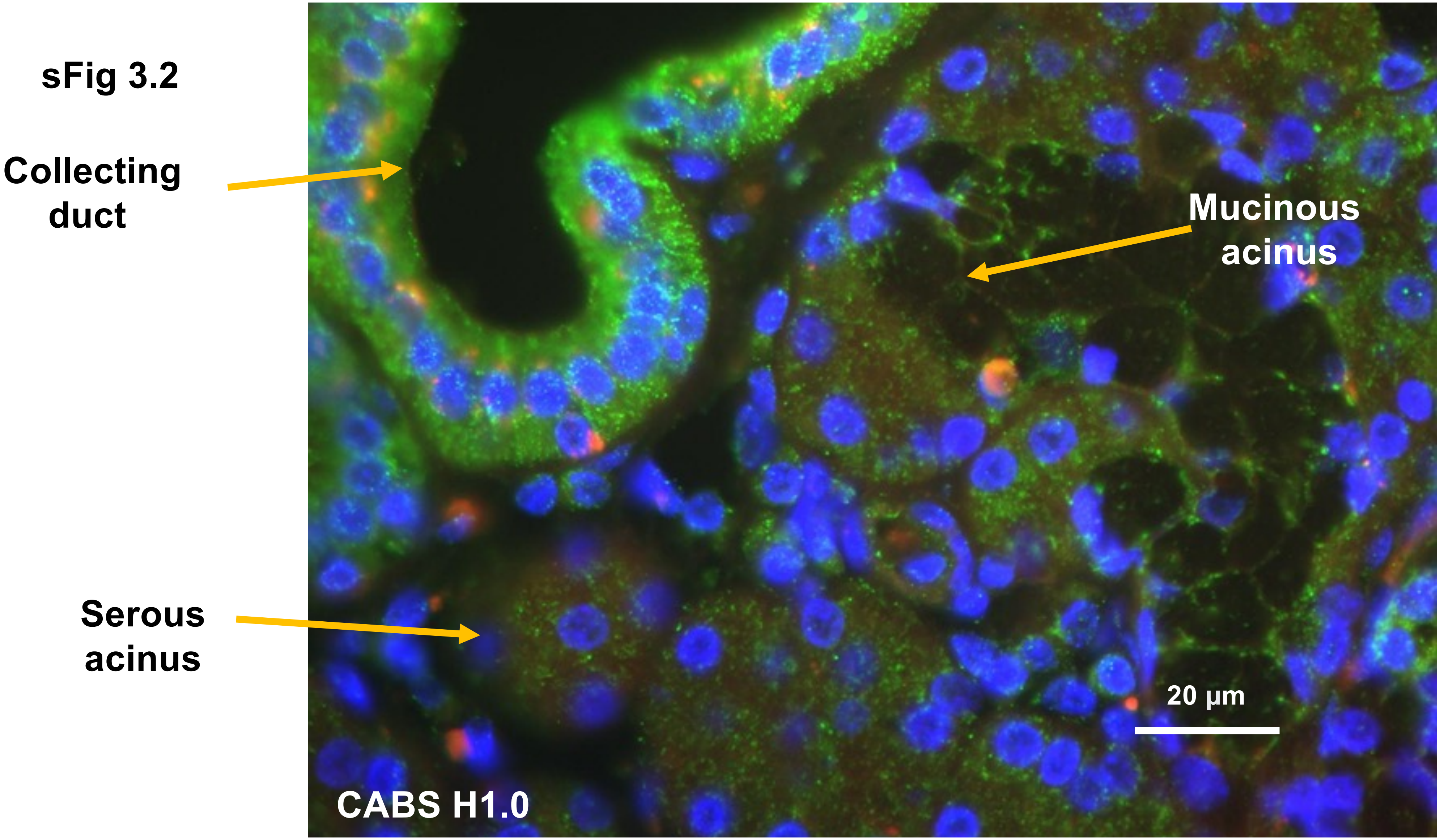

Collecting duct
Mucinous acinus
Serous acinus
20 µm
CABS H1.0

sFig 3.3

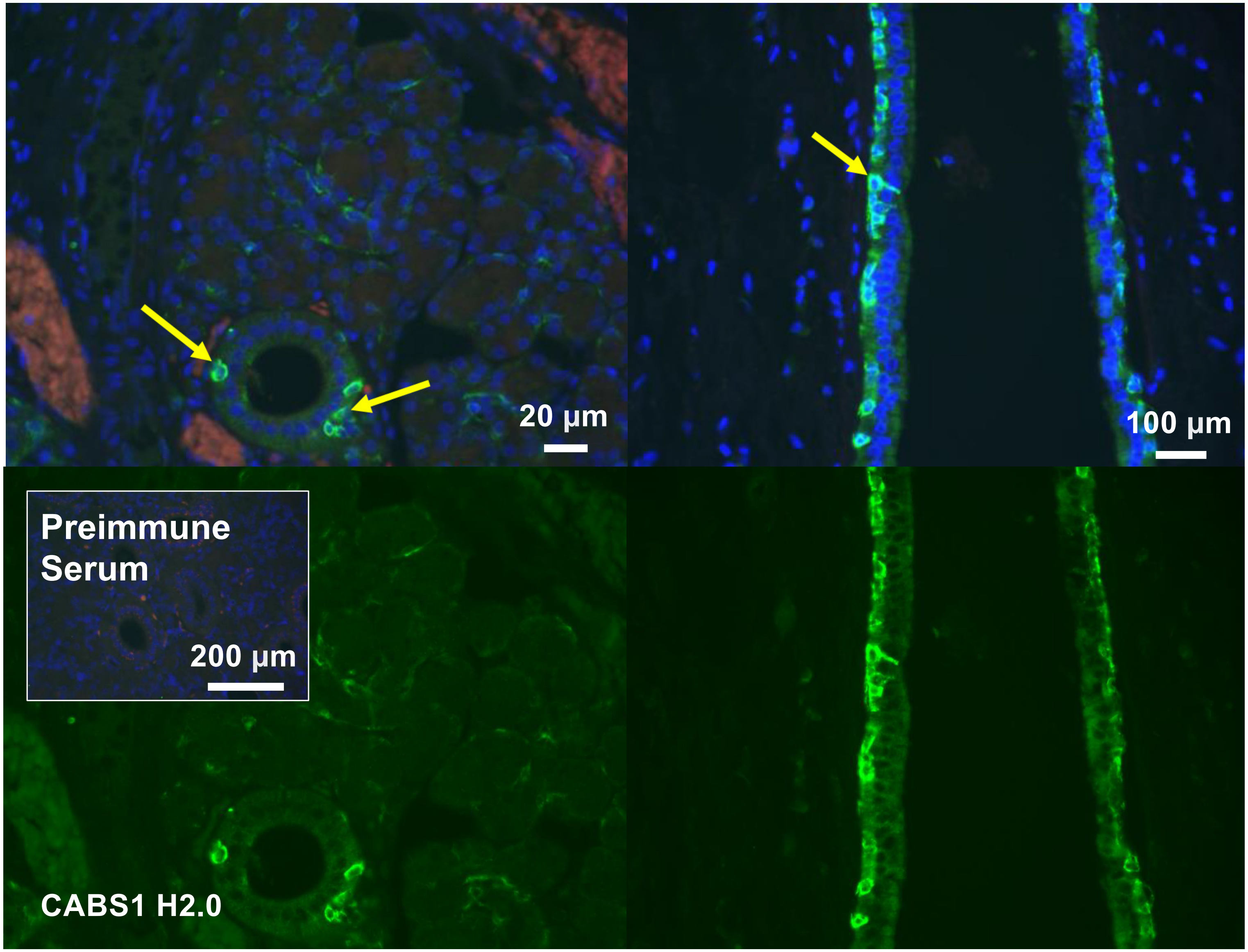

sFig 3.4

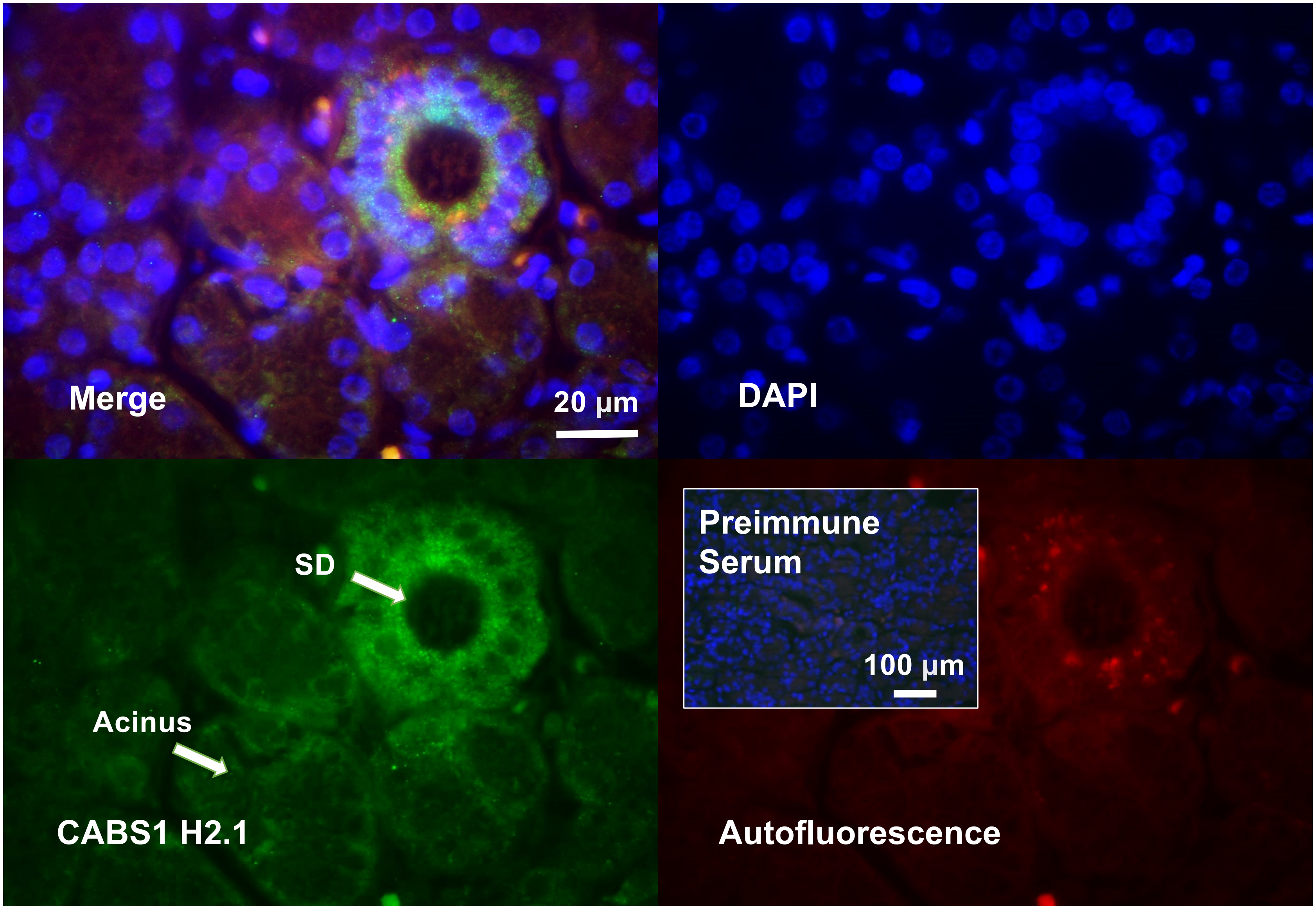
Merge
20 µm
DAPI
SD
Acinus
CABS1 H2.1
Preimmune
Serum
100 µm
Autofluorescence

sFig 3.5

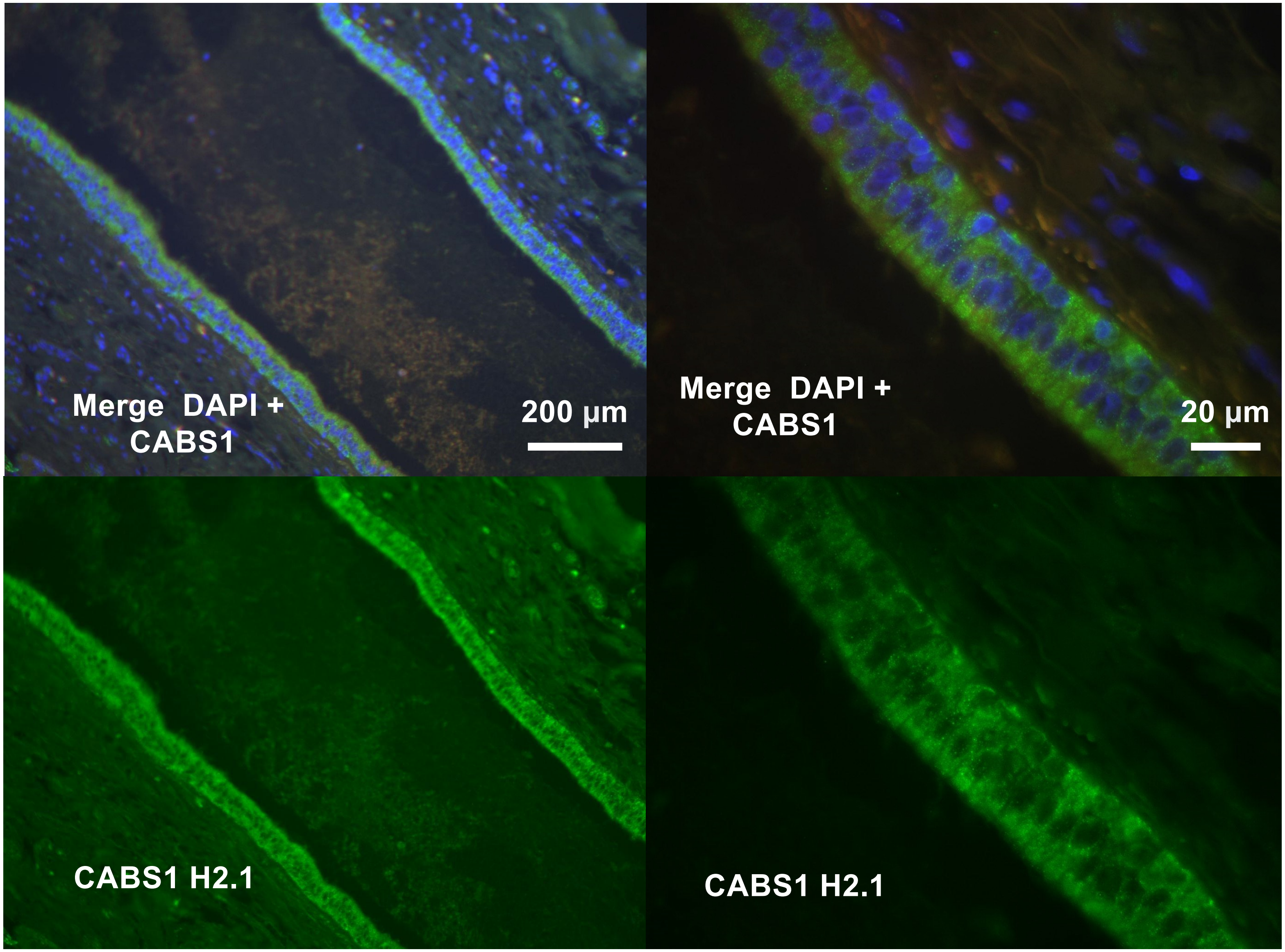
Merge DAPI +
CABS1
200 µm
Merge DAPI +
CABS1
20 µm
CABS1 H2.1
CABS1 H2.1

sFig 3.6

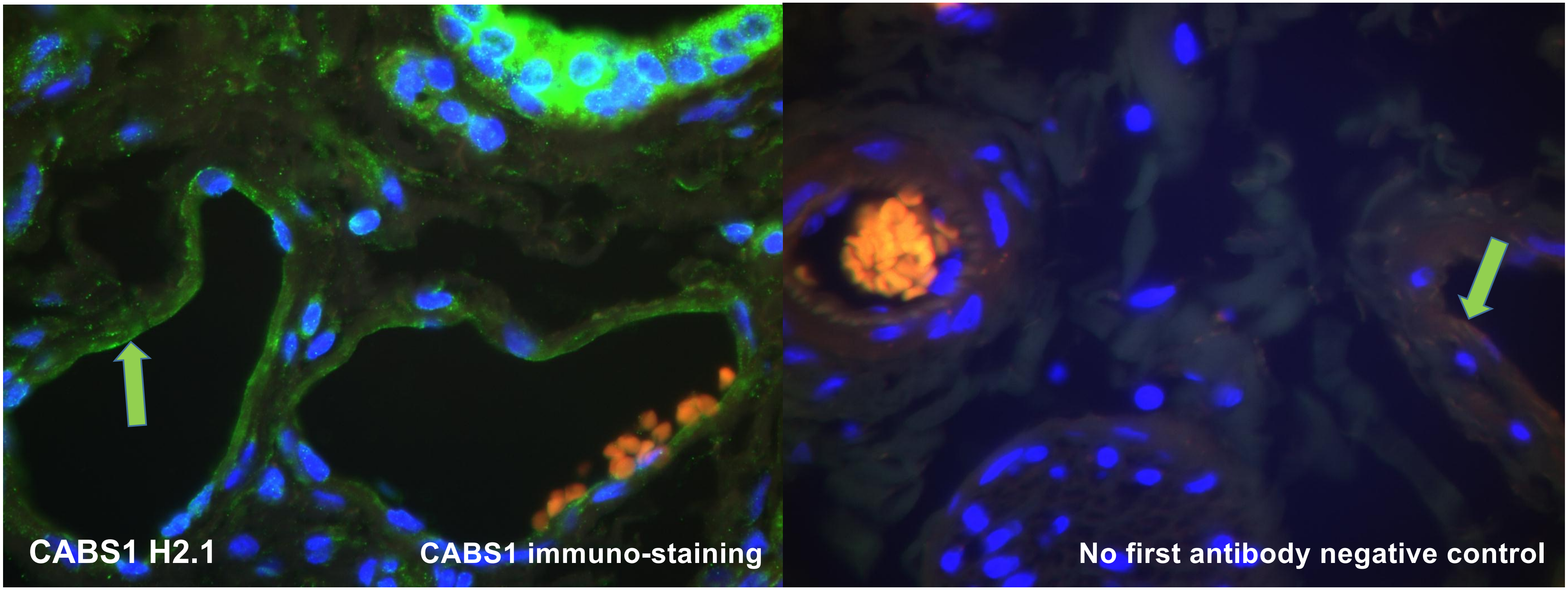
CABS1 H2.1
CABS1 immuno-staining
No first antibody negative control

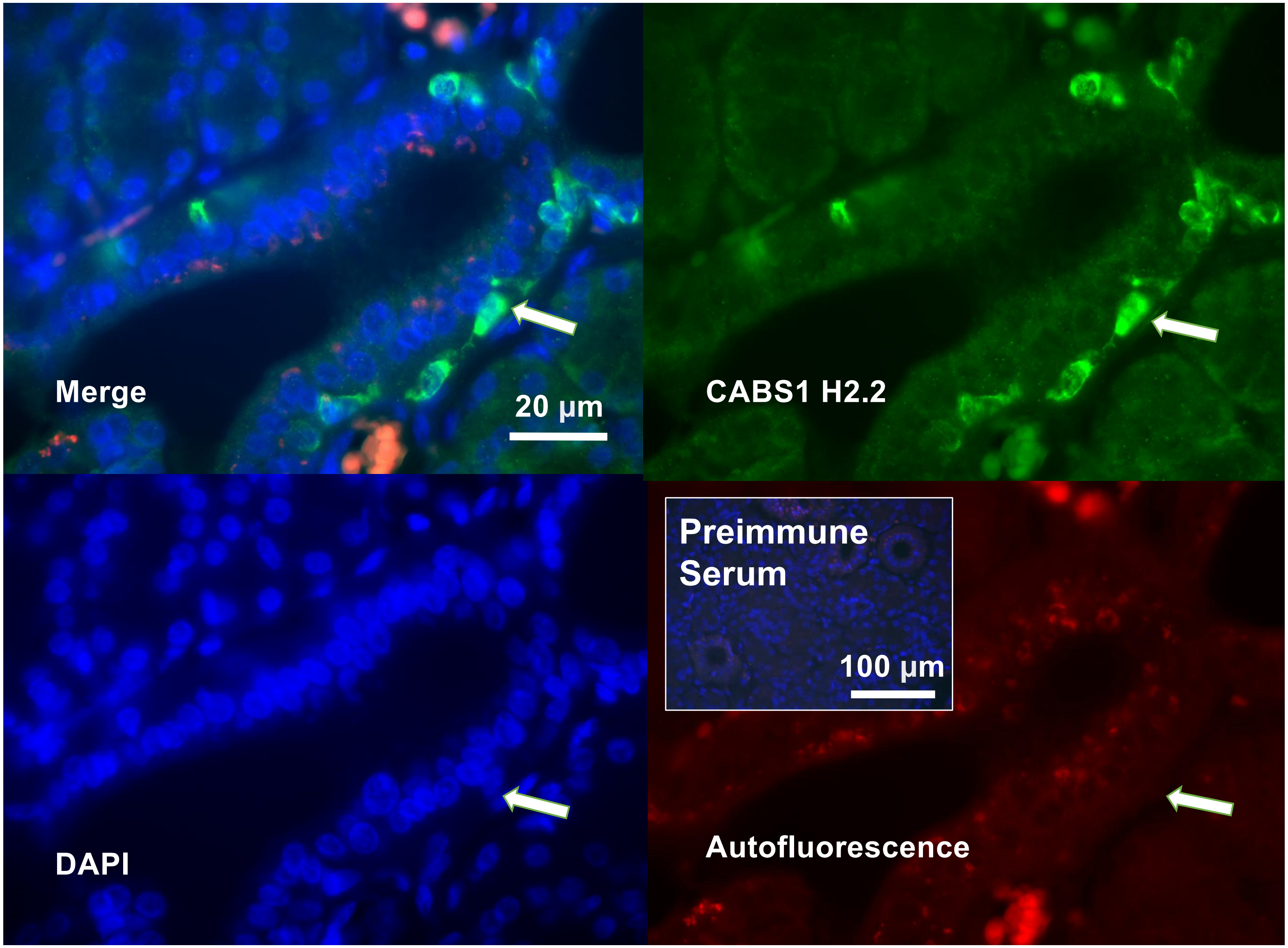
Merge
20 µm
CABS1 H2.2
DAPI
Preimmune
Serum
100 µm
Autofluorescence

sFig 3.7

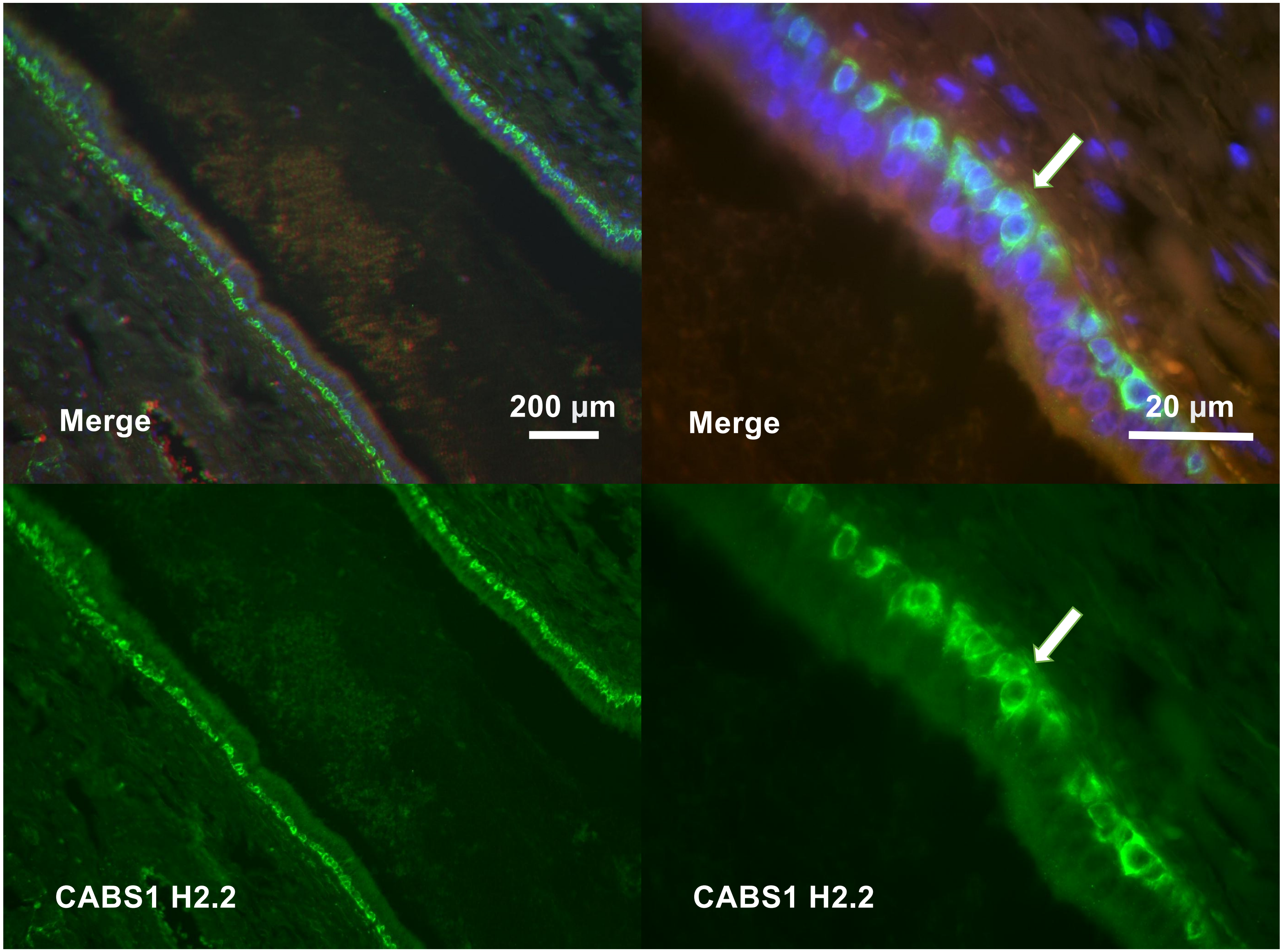

sFig 3.8
