# Supplementary material for: Identification and characterization of calcium binding protein, spermatid-associated 1 (CABS1)# in selected human tissues and fluids
Source: PLoS One. 2024 May 16;19(5):e0301855. doi: 10.1371/journal.pone.0301855 (PMC11098423; doi:10.1371/journal.pone.0301855)
Supplement: S1 Table — (PDF) [file pone.0301855.s004.pdf]

## Supplementary Table 1 - Glossary of Abbreviations

| | |
|---|---|
| **A** | artery |
| **AB** | abluminal cell of epithelium |
| **CABS1** | calcium binding protein, spermatid-associated 1 |
|     **hCABS1** | human |
|     **rhCABS1** | recombinant hCABS1 |
| **DS** | developing spermatocytes |
| **EC** | endothelial cell(s) |
| **ED** | excretory duct(s) |
| **GEO** | Gene Expression Omnibus |
| **GSK** | GlaxoSmithKline |
| **H&E** | hematoxylin and eosin |
| **L** | lobe(s) of salivary gland |
| **LC** | Leydig cell |
| **MA** | mucinous acinus/i |
| **mAb** | monoclonal antibody to CABS1, includes 15B11, 13G3 and 4D1 |
| **MS-seq** | mass spectroscopy sequencing |
| **N** | nerve |
| **NCIA** | nano-capillary immunoassay |
| **NCL** | negative control lysate, contains plasmid, not transfected with CABS1 |
| **OEL** | overexpression lysate of CABS1 |
| **pAb** | polyclonal antibody to CABS1, includes H1.0, 2.0, 2.1 and 2.2<br>spermatids |
| **PimmS** | Preimmune serum |
| **S** | |
| **SA** | serous acinus/i |
| **SC** | Sertoli cell |
| **SD** | salivary duct |
| **SG** | primary spermatogonia |
| **SM** | smooth muscle |
| **SMG** | submandibular gland |
| **SMR1** | submandibular rat 1 |
| **ST** | seminiferous tubule(s) |
| **TA** | tunica albuginea |
| **V** | vessel |
| **WB** | Western Blot |
