# Supplementary material for: Identification and characterization of calcium binding protein, spermatid-associated 1 (CABS1)# in selected human tissues and fluids
Source: PLoS One. 2024 May 16;19(5):e0301855. doi: 10.1371/journal.pone.0301855 (PMC11098423; doi:10.1371/journal.pone.0301855)
Supplement: S2 Table — (PDF) [file pone.0301855.s005.pdf]

Supplementary Table 2.

A) **Human CABS1 Western Blot samples for submandibular gland lysates (N = 8).**

Sample	Sex	Age at Collection
1	F	50
2	F	46
3	M	41
4	M	Unknown
5	F	59
6	M	64
7	F	65
8	M	63

B) **Human CABS1 immunohistochemistry samples for submandibular gland and testes tissue.**

<table>
<tr><th colspan="4">Case Western – Immunofluorescence</th></tr>
<tr><td rowspan="5">SMG</td><td>Diagnosis</td><td>Sex</td><td>Age at Collection</td></tr>
<tr><td>Squamous Cell Carcinoma</td><td>F</td><td>40</td></tr>
<tr><td>Squamous Cell Carcinoma</td><td>M</td><td>62</td></tr>
<tr><td>Sjogren’s Syndrome</td><td>Unknown</td><td>Unknown</td></tr>
<tr><td>Sjogren’s Syndrome</td><td>M</td><td>43</td></tr>
<tr><th colspan="4">University of Alberta – Immunoperoxidase Staining</th></tr>
<tr><td rowspan="3">SMG</td><td>Squamous Cell Carcinoma</td><td>M</td><td>69</td></tr>
<tr><td>Squamous Cell Carcinoma</td><td>F</td><td>73</td></tr>
<tr><td>Squamous Cell Carcinoma</td><td>F</td><td>62</td></tr>
<tr><td rowspan="4">Testes</td><td>Unknown Mass</td><td>M</td><td>58</td></tr>
<tr><td>Unknown</td><td>M</td><td>70</td></tr>
<tr><td>Unknown</td><td>M</td><td>73</td></tr>
<tr><td>Unknown</td><td>M</td><td>Unknown</td></tr>
</table>
