# Supplementary material for: Identification and characterization of calcium binding protein, spermatid-associated 1 (CABS1)# in selected human tissues and fluids
Source: PLoS One. 2024 May 16;19(5):e0301855. doi: 10.1371/journal.pone.0301855 (PMC11098423; doi:10.1371/journal.pone.0301855)
Supplement: S2 File — (PDF) [file pone.0301855.s007.pdf]

UNIVERSITY OF ALBERTA

ARISE
Alberta Research Information Services

Date: Tuesday, August 22, 2023 11:44:50 AM

Print Close

Table of Contents

Pro00001790

Packet Name: 2 - Smartform

- 1.1 Study Identification
- 1.2 Additional Approval
- 1.3 Funding Information
- 1.4 Conflict of Interest
- 1.5 Research Locations and Other Approvals
- 2.1 Study Objectives and Design
- 2.2 Research Methods and Procedures
- 2.9 Surveys and Questionnaires (including Online)
- 2.15 Use of Health Information
- 2.16 Clinical Trial
- 2.17 Data Safety and Monitoring for Clinical Trials
- 2.18 Collection of Human Biological Materials
- 2.21 Secondary Use of Human Biological Materials
- 3.1 Risk Assessment
- 3.2 Benefits Analysis
- 4.1 Participant Information
- 4.2 Additional Participant Information
- 4.4 Recruitment of Participants (non-Health)
- 4.5 Informed Consent Determination
- 5.1 Data Collection
- 5.4 Data Storage, Retention, and Disposal
- Documentation
- Final Page
- Add/Edit Funding Info without Manual Entry (ID00051380)
- Add/Edit Funding Info without Manual Entry (ID00066276)
- Add/Edit Funding Info without Manual Entry (ID00047813)
- Add/Edit Funding Info without Manual Entry (ID00041411)
- Add/Edit Funding Info without Manual Entry (ID00026319)
- Add/Edit Funding Info without Manual Entry (ID00053294)

Packet Name: 3 - Reviewer Notes

- Reviewer Notes

1.1 Study Identification

*All questions marked by a red asterisk * are required fields. However, because the mandatory fields have been kept to a minimum, answering only the required fields may not be sufficient for the REB to review your application.*

Please answer all relevant questions that will reasonably help to describe your study or proposed research.

1.0 * **Short Study Title** *(restricted to 250 characters)*:
Anti-inflammatory proteins and biomarkers of stress

2.0 * **Complete Study Title** *(can be exactly the same as short title)*:
Anti-inflammatory proteins and biomarkers of stress

3.0 * **Select the appropriate Research Ethics Board (***Detailed descriptions are available at here***):**
HREB Biomedical

4.0 * **Is the proposed research:**
Funded (Grant, subgrant, contract, internal funds, donation or some other source of funding)

5.0 * **Name of local Principal Investigator:**
A. Dean Befus

6.0 * **Type of research/study:**
Faculty/Academic Staff

7.0 **Investigator's Supervisor***(required for applications from undergraduate students, graduate students, post-doctoral fellows and medical residents to REBs 1 & 2. HREB does not accept applications from student PIs)*:

8.0 **Study Coordinators or Research Assistants:** People listed here can edit this application and will receive all email notifications for the study:

Name	Employer
There are no items to display	

9.0 **Co-Investigators:**People listed here can edit this application and will receive email notifications *(Co-investigators who do not wish to receive email, should be added to the study email list team below instead of here).*

Name	Employer
Marcelo Marcet-Palacios	MH Medicine

10.0 **Primary Admin Contact:**

11.0 **Study Team:***(Co-investigators, supervising team, other study team members) - People listed here cannot view or edit this application and do not receive email notifications.*

Last Name	First Name	Organization	Role/Area of Responsibility	Phone	Email
Serratos	Eduardo	University of Alberta	MSc student conducting the protein studies in saliva	780-492-3457	reyesser@ualberta.ca
Stickland	Mike	University of Alberta	Co-investigator	780-407-7144	mike.stickland@ualberta.ca
Gonshor	Aron	FL:UIDS iQ and GB Diagnostics	Co-investigator	514-941-4502	agonshor@fluidsiq.com
Buck	Robert	GB Diagnostics	Co-investigator	503-267-9965	geofluor@gmail.com
Ritz	Thomas	Southern Methodist University, Dallas, TX	Co-investigator	214-768-3724	tritz@mail.smu.edu
Puttagunta	Lakshmi	University of Alberta	Co-investigator		lputtagu@ualberta.ca
Peerani	Farhad	University of Alberta	Co-investigator		peerani@ualberta.ca
Canil	Sarah	Alberta Precision Labs	Co-investigator		sarah.canil@albertaprecisionlabs.ca
Ramielle Santos	Joy	University of Alberta	MSc Student working on CABS1		joyramie@ualberta.ca

ID: Pro00001790 **Pro00001790** 1.2 Additional Approval
Status: Approved

1.2 Additional Approval

1.0 * **Departmental Review:** ***Please note only ONE Department Review is required. Please ensure that this section reflects only the PRIMARY Department of the study PI.***

MH Medicine

2.0 **Internal Review** *(If the Principal Investigator is in the Department of Medicine complete the Department of Medicine Request for Internal Approval form and upload it to the "Documentation" section of this application under item 11.0 "Other Documents". Note that all fields in the*

form are required. The form is available at here):

Medicine

ID: Pro00001790 **Pro00001790** 1.3 Funding Information
Status: Approved

1.3 Study Funding Information

1.0 * **Type of Funding:**
Grant (external)
Contract (eg. Commercial, Industry, For-profit funding, etc)

* **Principal Investigator Contact Name***(this is the name of the person at your site locally who is responsible for receiving study invoices):*
See legacy file

Position/Title:

* **Office Address** *(Office Address, City, Province or State, Postal Code)*:
See legacy file

* **Phone Number** *(area code, phone number, extension)*:

* **Email:**

2.0 * **Indicate which office administers your award.** *(It is the PI's responsibility to provide ethics approval notification to any office other than the ones listed below)*
University of Alberta - Research Services Office (RSO)

To connect your ethics application with your funding: provide all identifying information about the study funding – multiple rows allowed. For Project ID, enter a Funding ID provided by RSO/PeopleSoft Project ID*(for example, RES0005638, G018903401, C19900137, etc).***Enter the corresponding title for each Project ID.**

	Project ID	Title	Grant Status	Sponsor	Project Start Date	Project End Date	Purpose	Other Information
View	RES0048542							Principal Investigator Dr. Marcelo Marcet 2020 - 2025-
View	RES0058116	Gen Res - Befus,A. Dean	Submitted	General Research Project (PI)	3/1/2022	3/31/2025	General Research	
View	RES0044356							2019-2022

	Project ID	Title	Grant Status	Sponsor	Project Start Date	Project End Date	Purpose	Other Information
View	G600000410							2005 - 2019
View	RES0031659							2016-2019
View	RES0051018							This is a supplementary fund for Dr. Marcelo Marcet (NSERC)

3.0 * **Funding Source**

3.1 Select all sources of funding from the list below:

Network of Centres of Excellence	NCE
NSERC - Natural Sciences And Engineering Research Council	NSERC

3.2 If your source of funding is not available in the list above, click "Add" below and write the Sponsor/Agency name(s) in the free text box that pops up.*(Nore: You may reflect multiple sources of funding by continuing to click "Add" to add each additional source of funding).*

GBD Inc. RES0044356

4.0 * **Indicate if this research sponsored or monitored by any of the following:**

Not applicable

The researcher is responsible for ensuring that the study complies with the applicable US regulations. The REB must also comply with US Regulations.

ID: Pro00001790 **Pro00001790** 1.4 Conflict of Interest
Status: Approved

1.4 Conflict of Interest

1.0 * **Are any of the investigators or their immediate family receiving any personal remuneration (including investigator payments and recruitment incentives but excluding trainee remuneration or graduate student stipends) from the funding of this study that is not accounted for in the study budget?**

○ Yes ● **No**

2.0 * **Do any of investigators or their immediate family have any proprietary interests in the product under study or the outcome of the research including patents, trademarks, copyrights, and licensing agreements?**

● **Yes** ○ No

3.0 * **Is there any compensation for this study that is affected by the study outcome?**

◯ Yes ⬤ **No**

4.0 *** Do any of the investigators or their immediate family have equity interest in the sponsoring company? (This does not include Mutual Funds)**

◯ Yes ⬤ **No**

5.0 *** Do any of the investigators or their immediate family receive payments of other sorts, from this sponsor (i.e. grants, compensation in the form of equipment or supplies, retainers for ongoing consultation and honoraria)?**

◯ Yes ⬤ **No**

6.0 *** Are any of the investigators or their immediate family, members of the sponsor's Board of Directors, Scientific Advisory Panel or comparable body?**

◯ Yes ⬤ **No**

7.0 *** Do you have any other relationship, financial or non-financial, that, if not disclosed, could be construed as a conflict of interest?**

◯ Yes ⬤ **No**

Please explain if the answer to any of the above questions is Yes:
Dr. Befus has filed a patent on CABS1 in stress in collaboration with TEC Edmonton, the University of Alberta, two laboratory workers (have left the laboratory) and Dr. Ritz from Southern Methodist University. The patent is approved. Dr. Befus, with the help of TEC Edmonton and University of Alberta has a Licensing Agreement with GB Diagnostics (identified collaborators).

Important

If you answered YES to any of the questions above, you may be asked for more information.

ID: Pro00001790 **Pro00001790**
Status: Approved 1.5 Research Locations and Other Approvals

1.5 Research Locations and Other Approvals

1.0 *** List the locations of the proposed research, including recruitment activities. Provide name of institution, facility or organization, town, or province as applicable**

As done previously in this project, I will collect up to five human salivary gland samples that had been acquired at surgery and that would otherwise be discarded (in collaboration with Dr. Lakshmi Puttagunta, Pathologist). Samples were acquired from surgeries performed at the U of A and at the Royal Alexandra Hospital. They will be used to create an external standard for the CABS1 proteins for our semi-quantitative analyses of CABS1 in the saliva samples from Drs. Ritz, Cernak and Peerani.
We also have permission to acquire human blood and salivary samples to study the potential endocrine and exocrine secretion of anti-inflammatory proteins/peptides from salivary glands and other possible sources.
In our collaboration with FLUIDS iQ and GB Diagnostics we have begun to focus on serum samples as these are more easily standardized for

biomarker analyses. Our initial focus is on 200 samples from subjects with chronic obstructive lung disease (COPD) in collaboration with Dr. Mike Stickland (Pro00038838).
Our studies with salivary samples will be delayed while our focus is on serum samples and COPD. Samples of human saliva will then be assayed. Saliva samples have acquired from ethically approved studies conducted by our colleagues in Southern Methodist University, Dallas, Tx (Dr. Thomas Ritz) and in U of A (Dr. Ibolja Cernak and Dr. Peerani). The samples from Dallas are from a replication of a study of human stress associated with final examinations; we are testing if this examination stress modifies secretion of putative anti-inflammatory peptides from the salivary glands and identifies these forms of CABS1 protein as biomarkers of stress. The previous studies received U of A approval through an amendment to this protocol in 2012-2013.
The saliva samples from Dr. Cernak (Pro00035703, U of A) were collected from military personnel in Canada and Afghanistan. Samples from Dr. Peerani (Pro00079377) are of a stress intervention study in Ulcerative Colitis. I will conduct secondary analyses of CABS1 in these samples in my research laboratory in HRMC and will be blinded to any sample identifiers.

Saliva samples for the development of an internal standard are collected from laboratory personnel (volunteers).

2.0 * **Indicate if the study will use or access facilities, programmes, resources, staff, students, specimens, patients or their records, at any of the sites affiliated with the following** *(select all that apply):*

Alberta Health Services Institutions and Facilities
Covenant Health Institutions and Facilities
Capital Care Institutions and Facilities

List all health care research sites/locations:
Surgical specimens of human salivary glands that would otherwise be discarded will be collected through AHS resources at the UofA hospital and potentially the Royal Alexandra Hospital, Edmonton. All saliva samples for secondary analyses will be collected by my collaborators at U of A or other Universities/institutions and sent to us. We will collect a limited number of saliva samples from volunteers in our lab to create a saliva pool to use as an external standard for our secondary analyses of samples from collaborators. COPD samples have been acquired through several facilities in the Edmonton zone, including Covenant Health.

3.0

Multi-Institution Review

* **3.1 Has this study already received approval from another REB?**
○ **Yes** ● **No**

4.0 **If this application is closely linked to research previously approved by one of the University of Alberta REBs or has already received ethics approval from an external ethics review board***(s)***, provide the study number, REB name or other identifying information. Attach any external REB application and approval letter in the Documentation Section – Other Documents.**
Resilence Enhancement in Military, (Pro00035703); Dr. Ibolja Cernak, U of A. Also Pr00066348.

Dr. Farhad Peerani, U of A Pro00079377, Stress and Ulcerative Colitis.

Smartphone Monitoring of Airway Inflammation during Psychological Stress in Asthma,

IRB Application #2015-109-RITT; Southern Methodist University, Dr. Thomas Ritz.

Dr. Mike Stickland; COPD. Pro00038838.

2.1 Study Objectives and Design

1.0 * **Provide a lay summary of your proposed research which would be understandable to general public**

In studies in an experimental model of inflammatory disease in rats, we have identified an anti-inflammatory pathway regulated by the nervous system (a mind-body pathway?). Interestingly, the source of the anti-inflammatory activity is in the salivary glands.

An important research priority was whether or not this anti-inflammatory pathway exists in humans and if human salivary glands express the same gene or at least a closely related gene and whether or not it has anti-inflammatory activity. We discovered that the human does not have the same gene as in the rat, but we have identified a related gene (CABS1) and established that a fragment from the protein can reduce inflammation. Moreover, to study if this pathway is under the control of the nervous system, we tested if stress in humans would change the levels of this protein in saliva. We discovered that this is true and identified that stress enhances the levels of several forms of the protein. Indeed some smaller forms of the protein appear to identify individuals who are resistant to some of the effects of stress; they are resilient.

We are now working with serum samples from subjects with COPD and normal controls in collaboration with Dr. Mike Stickland, FLUIDS iQ and GB Diagnostics. We have promising resutls that suggest our biomarker panel may help identify disease activity in COPD and help assess risk of exacerbations.

Once the CABS1 stress biomarker panel analyses have been completed for COPD, we will then return to our earlier focus on human saliva samples through continued collaborations with Dr. Ritz, a psychologist in Dallas, TX and through collaborations with Dr. Ibolja Cernak, at U of A in her studies of stress and resilience in Canadian military personnel. Currently, saliva samples are also being collected to study CABS1 levels associated with stress reduction approaches in participants with ulcerative colitis (collaborator Dr. Farhad Peerani).

Future work could involve determining whether psychosocial or other interventions might enhance this mind-body pathway to reduce stress and some of its effects on human health.

2.0 * **Provide a full description of your research proposal outlining the following:**

- **Purpose**
- **Hypothesis**
- **Justification**
- **Objectives**
- **Research Method/Procedures**
- **Plan for Data Analysis**

The research in my laboratory is focused on the analysis of CABS1 proteins in saliva samples collected by my collaborators during their studies of stressful situations in humans. We have established that CABS1 is expressed in human salivary glands (St. Laurent et al., 2015, attached) and that in three independent studies (baseline, acute stress and chronic stress), CABS1 levels in saliva can be modified during stress (Ritz et al., in preparation; attached). As a result of these discoveries, we have filed a provisional patent through TEC Edmonton, and are working towards further validation of our discoveries and extend of the results and their implications. All of my work in this ethics amendment involves secondary analyses of saliva samples collected by my colleagues under the approval of their ethics applications.

Recently, our focus has become studies of serum samples in subjects with COPD (from Dr. Stickland) and normal controls with our stress biomarker panel. In addition to measuring CABS1 levels and its possible proteolytic fragments, we have also included measures of oxidative stress and anti-oxidants (peroxidase activity in this panel. We have promising results in our initial studies and will expand and validate these investigations to determine if our biomarker panel can accurately assess disease activity and risk for exacerbations; both could be valuable clinical resources. We are currently working to submit an application to the Genome Canada Genomes Application Partnership Program (GAPP) to support these studies.

Our grant from AllerGen NCE Inc that supported the initial work on CABS1 and psychological stress has recently ended. Two manuscripts were (2017, 2019) published with this funding and have extended our 2015 paper that described our discovery of CABS1 in human salivary glands and documented its anti-inflammatory activity.

Dr. Thomas Ritz our colleague who is an experimental psychologist in Dallas, TX, has completed the replication our previous study of chronic, final examination stress. Participants with asthma and healthy controls (each group n=30-40) were included. Saliva was collected together with inflammatory markers, lung function, and cardiorespiratory activity (including gas exchange). The saliva samples will be sent to my laboratory and together with FLUIDS iQ and GB Diagnostics analyses completed. Thereafter, we plan to begin the analyses of the saliva samples from Dr. Cernak's study of Reslience Enhancement in Military (REIM) study. REIM was designed to examine resilience, stress and cognitive changes in a cohort of personnel across three assessment points: 1) during pre-deployment training (B1, n = 116), 2) during deployment to Afghanistan (B2, n = 123) and 3) within 6-9 months of return to Canada, in the post-deployment reintegration phase (B3, n = 78). At each time, a comprehensive package of assessment was conducted, including saliva collection, questionnaires to examine patient history and psychosocial stress, and advanced cognitive testing using the Cambridge Cognition Automated Test Battery (CANTAB). REIM involved dynamic enrolment, and includes soldiers at each baseline who did not necessarily complete other baselines due to non-deployment or recruitment to the study after B1. Therefore, numbers of soldiers who completed both B1 & B2 (n = 91) and who completed all three baselines, i.e.B1, B2 & B3 (n = 51) differ from number of soldiers enrolled in each baseline. Whole saliva samples were collected in the morning at least 2 h after meals and prior to daily training. Participants rinsed their mouths with water 10 min before saliva collection. Saliva was collected by the passive drool technique into a saliva collection aid attached to a 2 mL sterile cryovial. Participants recorded the time to fill three sequential cryovials, which were stored on ice until return to the laboratory, and then sent to the Canadian Biosample Repository (-80°C) at UA. One saliva sample from each subject and time point will be available for our studies of CABS1 levels and our results will be provided to Dr. Cernak for studies of their relationships to resilence, stress and cognitive changes in the military personnel.

Studies with Dr. Peerani will also involve secondary analyses of CABS1 levels done on samples collected by Dr. Peerani and his team (Pro00079377). This is a study of a stress intervention in ulcerative colitis patients at U of A.

Studies of Drs. Stickland, Ritz, Cernak and Peerani have received ethical approval from their respective institutions.

3.0 **Describe procedures, treatment, or activities that are above or in addition to standard practices in this study area** *(eg. extra medical or health-related procedures, curriculum enhancements, extra follow-up, etc)***:**
NA

4.0 **If the proposed research is above minimal risk and is not funded via a competitive peer review grant or industry-sponsored clinical trial, the REB will require evidence of scientific review. Provide information about the review process and its results if appropriate.**
My studies of CABS1 in the saliva samples from Drs. Ritz and Cernak is funded by the Allergy Genes and Environment Network of Centres of Excellence (AllerGen NCE), Canada. Within AllerGen, the review was conducted by both the Network Supported Intellectual Properties Committee and by the Research Management Committee.
The studies of stress in ulcerative colitis is funded by a grant from the University Hospital Foundation to Dr. Peerani.
Studies of COPD samples are funded in part by my Transition to Retirement Account (active until June 30, 2021, as well as in-kind support from FLUIDS-iQ and GB Diagnostics (partners). A Genome Canada application is pending review for this work as well.

5.0 **For clinical trials, describe any sub-studies associated with this Protocol.**
For the COPD study samples from the Clinical Trial are being analysed for levels of CABS1 and associated oxidative stress.

2.2 Research Methods and Procedures

Some research methods prompt specific ethical issues. The methods listed below have additional questions associated with them in this application. If your research does not involve any of the methods listed below, ensure that your proposed research is adequately described in Section 2.1: Study Objectives and Design or attach documents in the Documentation Section if necessary.

1.0 * **This study will involve the following***(select all that apply)*
Surveys and Questionnaires (including internet surveys)
Clinical Trial
Collection of Human Biological Materials (ie. blood, tissue etc.)
Use of Health Information - *See NOTE 1 below*
Secondary Use of Human Biological Materials - *See NOTE 2 below*

NOTE 1: Select this if you are directly collecting health information as part of your protocol OR will be conducting a chart/record review/reviewing health data secondarily. This includes anonymized or identifiable health information.

NOTE 2: Select this option if this research ONLY involves analysis of blood/tissue/specimens originally collected for another purpose but now being used to answer your research question. If you are enrolling people into the study to prospectively collect specimens to analyze you SHOULD NOT select this box.

NOTE 3: This section is intended to reflect the secondary use of non-health data. Do NOT select this if you are using data that originally came from health sources, i.e., anonymized administrative data.

ID: Pro00001790 **Pro00001790**
Status: Approved 2.9 Surveys and Questionnaires (including Online)

2.9 Surveys and Questionnaires (including Online)

1.0 **How will the survey/questionnaire data be collected (i.e. collected in person, or if collected online, what survey program/software will be used etc.)?**
This is covered under the ethics approval for Dr. Thomas Ritz, Southern Methodist University, Dallas, TX. None of the information will be available in our laboratory in University of Alberta. We will receive the samples in a blinded fashion and together with FLUIDS iQ and GB Diagnostics, will analyze the biomarkers in the samples. The sample values will be returned to Dr. Ritz for unblinding and analyses in relation to the questionnaire and survey information.
Dr. Stickland (Clinical Trial.gov) research protocol attached for COPD subjects.

2.0 **Where will the data be stored once it's collected (i.e. will it be stored on the survey software provider servers, will it be downloaded to the PI's computer, other)?**
The information will be stored with Dr. Ritz in Southern Methodist University. In U of A we will have not access to the individual survey or questionnaire information.
Dr. Stickland has the data on the COPD subjects. We are doing biomarker assays in a manner blinded to the subject information, and Dr. Stickland will unblind the data and do the analyses of association with clinical information.

3.0 **Who will have access to the data?**
Dr. Ritz and his colleagues in Dallas.

Dr. Stickland has the COPD health information.

4.0 **If you are using a third party research tool, website survey software, transaction log tools, screen capturing software, or masked survey sites, how will you ensure the security of data gathered at that site?**

ID: Pro00001790 **Pro00001790** 2.15 Use of Health Information
Status: Approved

2.15 Use of Health Information

For guidance on completing an application seeking to conduct a review of records containing personally identifiable health information, please review the following guidance: https://www.ualberta.ca/research/research-support/research-ethics-office/human-research-ethics/information-and-data/health-information/chart-review.html

1.0 **Estimate the number of records you will review/receive** *(ie. We will review approximately 300 charts, we will receive approximately 3000 patient records from the data custodian)*
This information is covered by Dr. Stickland's ethics (Pro00038838). The 200 serum samples are from
93 subjects (93 charts).
My laboratory will have no access to the charts.

2.0 **List ALL of the data source(s) that you will be using to get your data***(ie. Paper charts, e-clinician, DIMR records, NetCare, PAC system etc.)*

3.0 **Will the chart/record review be:**
RETROSPECTIVE: The dates of the records that will be reviewed do not exceed the date of this ethics application

4.0 **Provide the start and end date of the records you will review***(Note: these dates do NOT refer to when the review will be performed but the actual dates on the medical records, ie., we need administrative data from January 1, 2000 to December 31, 2010):*

Start Date:

End Date:

5.0 **Will individual consent be sought?**
○ Yes ○ No

6.0 **How will the data be received?**

If you are conducting a secondary review of health data please remember to upload the following to the Documentation Section:

1. *Your data collection sheets or a listing of the variables that you wish to collect.*
2. *If you are collecting health data using AHS or Covenant Health resources, you will be required to upload a formal research proposal/protocol to the Documentation Section*

2.16 Clinical Trial

1.0 **Protocol**

1.1 Protocol Number (if applicable):
Pro00038838 (Dr. Stickland)

1.2 Clinical trials must be registered before participant recruitment can begin. Provide registry and registration number, e.g. clinicaltrials.gov:
Clinicaltrials.gov NCT01949727 (Dr. Stickland trial)

2.0 **Is this an investigator-initiated clinical trial?**

* **Is this study authored and initiated by a researcher from the University of Alberta, Alberta Health Services and/or Covenant Health?**
○ Yes ● **No**

* **Is this study authored or sponsored by any outside entity including, but not limited to, a pharmaceutical company or clinical research organization?**
● **Yes** ○ No

3.0 ***Does the study involve any of the following?**

Answer	Description
○ Yes ● **No**	A drug, device, biologics, vaccine or natural health product not marketed in Canada?
○ Yes ● **No**	A comparative bioavailability trial?
○ Yes ● **No**	Use of a marketed drug, device, biologics, vaccine, or natural health product outside the parameters of its officially “approved use” by Health Canada?

If you have answered yes to any of the questions above, a Health Canada Clinical Trial Application (CTA) may be required. The investigator MUST coordinate with the University of Alberta - Quality Management in Clinical Research for all Health Canada clinical trials, as the University will be the named Sponsor of the trial. Please contact lori.anderson@ualberta.ca for assistance.

4.0 **Trial Phase:**
There are no items to display

5.0 **Describe the provisions made to break the code of a double-blind study in an emergency situation, and indicate who has the code (if applicable):**

6.0 **Provide justification for using placebo or no-treatment arm (if applicable):***(i.e. why/how is it OK to give a participant an inactive substance instead of a treatment)*

7.0 **Describe the clinical criteria for withdrawing an individual participant from the study due to safety or toxicity concerns (if applicable):**

8.0 * **Expected Length/Duration of Clinical Trial***(in months)***:**
49

2.17 Data Safety and Monitoring for Clinical Trials

1.0 * **Check one that most accurately reflects the plan for data safety and monitoring for this study:**
The study will be monitored only by the study investigators.

2.0 * **Describe data monitoring procedures while research is going on. Include details of planned interim analysis, Data Safety Monitoring Board, or other monitoring systems:**
See legacy file

3.0 * **Summarize any pre-specified criteria for stopping or changing the study protocol due to safety concerns:**
See legacy file

2.18 Collection of Human Biological Materials

1.0 * **Indicate the human biological material(s) that will be collected** *(for example, blood, urine, CSF, liver tissue, etc.)***:**
We will study previously collected saliva, but have also collected samples in our lab for studies to optimize collection, storage and CABS1 analyses.

The protocol has previously included blood and saliva that we might collect and we wish this to remain active. However, until we have new data, we have no specific plans for additional blood studies.

2.0 * **Specify all intended uses of collected specimen:**
Saliva samples will be used for analyses of CABS1 proteins, primary using Western blot or Wes-TM assays.

3.0 * **This study will involve the following** *(select all that apply)***:**
Collection of sample for immediate use
Collection of sample for banking (future use)
Secondary analysis of sample previously collected for clinical or research purposes

4.0 **Explain how and by whom the specimen will be collected**
Some samples are collected by Mr Reyes-Serratos in our laboratory for studies of optimization of CABS1 recovery, storage and analyses.
All the samples for secondary analyses are collected by our collaborators, Drs. Stickland Ritz, Cernak and Peerani.

5.0 **Explain HOW the specimen will be stored:**
Once in my lab, the specimens will be stored at -20 to -80C, depending on the duration.

Dr. Stickland's samples were stored in his -80C freezer (his lab).

6.0 **Explain WHERE the specimens will be stored** *(e.g. include information if the specimens will be sent out of the province)***:**
In Rm 569 HMRC in -80C freezer once that we have received the samples

from Drs Ritz, Peerani and from the Biorepository of Dr. Cernak.
Dr. Stickland's samples were stored in his laboratory.

7.0 **Explain HOW LONG the specimens will be stored:**
Specimens will be stored until the analyses in completed. If a portion of the specimen remains, this will be stored for several years until utilized. Some of our original predictions of timelines have been extended as we have worked to improve our immunoassays by both developing highly specific monoclonal antibodies and also by developing a collaboration with a commercial diagnostic company with outstanding expertise in immunoassay develop with high sensitivity and specificity.

2.21 Secondary Use of Human Biological Materials

1.0 **Outline where will you be getting the human biological materials from?**
Saliva samples for secondary analyses of CABS1 will be received from Drs. Ritz (Southern Methodist University, Dallas, TX), Cernak and Peerani (serum samples also), U of A.
Serum samples from COPD subjects are from Dr. Stickland, U of A.

2.0 **How/under what authority were these human biological materials originally collected?***(i.e. clinical specimens now being used for research, collected under a previous research protocol)*
Each of my colleagues have collected the samples following ethical approval from their respective institutions:

Dr. Ritz: IRB #2015-109-RITT (Southern Methodist University)
Dr. Cernak: Pro00035703 and Pro00066348
Dr. Peerani: Pro00079377
Dr. Stickland (Pro00038838)

3.0 **If specimens were originally collected under a research protocol, please outline how the proposed use of the samples is consistent with the parameters or restrictions of use described at the time of initial collection***(i.e. consent for future use was outlined in original consent form or ethics approval documentation)*
All approvals indicated that the samples were to be stored for future analyses of salivary or serum biomarkers.

4.0 **Are the human biological materials you will be receiving/using:**
Non-identifiable (i.e. you will not receive any identifiable health information linked to the specimens, nor would you ever be able to identify who the specimen came from)

3.1 Risk Assessment

1.0 *** Provide your assessment of the risks that may be associated with this research:**
Minimal Risk - research in which the probability and magnitude of possible harms implied by participation is no greater than those encountered by participants in those aspects of their everyday life that relate to the research (TCPS2)

2.0 *** Select all that might apply:**

	Description of Possible Physical Risks and Discomforts
No	Participants might feel physical fatigue, e.g. sleep deprivation
No	Participants might feel physical stress, e.g. cardiovascular stress tests
No	Participants might sustain injury, infection, and intervention side-effects or complications
No	The physical risks will be greater than those encountered by the participants in everyday life

	Possible Psychological, Emotional, Social and Other Risks and Discomforts
No	Participants might feel psychologically or emotionally stressed, demeaned, embarrassed, worried, anxious, scared or distressed, e.g. description of painful or traumatic events
No	Participants might feel psychological or mental fatigue, e.g intense concentration required
No	Participants might experience cultural or social risk, e.g. loss of privacy or status or damage to reputation
No	Participants might be exposed to economic or legal risk, for instance non-anonymized workplace surveys
No	The risks will be greater than those encountered by the participants in everyday life

3.0 *** Provide details of all the risks and discomforts associated with the research for which you indicated YES or POSSIBLY above.**
There are no risks associated with the studies to be conducted in my lab in the current proposal. The human samples are complete and I am doing biochemical analyses of CABS1 protein and fragments in these samples. There is no linkage to patient identification; all sample analyses is to be done in a blinded fashion to any subject identifiers.

4.0 *** Describe how you will manage and minimize risks and discomforts, as well as mitigate harm:**
Saliva collection by my collaborators has minimal risk.

5.0 **Is there a possibility that your research procedures will lead to unexpected findings, adverse reactions, or similar results that may require follow-up** *(i.e. individuals disclose that they are upset or distressed during an interview/questionnaire, unanticipated findings on MRI, etc.)***?**

○ Yes ○ No

6.0 **If you are using any tests in this study diagnostically, indicate the member(s) of the study team who will administer the measures/instruments:**

Test Name	Test Administrator	Organization	Administrator's Qualification

There are no items to display

7.0 **If any research related procedures/tests could be interpreted diagnostically, will these be reported back to the participants and if so, how and by whom?**
NA

3.2 Benefits Analysis

1.0 * **Describe any potential benefits of the proposed research to the participants. If there are no benefits, state this explicitly:**
No benefits to the subjects - research uses tissue and samples that would otherwise not be collected or would be discarded.

2.0 * **Describe the scientific and/or scholarly benefits of the proposed research:**
These studies may further our knowledge of a novel anti-inflammatory pathway in humans under mind-body controls. Such a pathway might be a component of the placebo effect in humans and thus efforts to understand its characteristics and controls could be of great scientific and medical benefit. Our efforts to evaluate levels of CABS1 protein/fragments have uncovered that these appear to be novel biomarkers of this pathway and as associated with stress and perhaps resilience to stress. Further study may allow us to be monitor the activities of this pathway and to modulate these through various psychosocial or pharmacological interventions.

3.0 **If this research involves risk to participants explain how the benefits outweigh the risks.**
Given the low risk, and the potential benefits, the benefit side of the ratio is favorable.

4.1 Participant Information

1.0 * **Will you be recruiting human participants** *(i.e. enrolling people into the study, sending people online surveys to complete)***?**
● **Yes** ○ No

1.1 Will participants be recruited or their data be collected from Alberta Health Services or Covenant Health or data custodian as defined in the Alberta Health Information Act?
○ Yes ● **No**

1.2 Would you like to include information about this study on the Be The Cure searchable database?

○ Yes ● **No**

ID: Pro00001790 **Pro00001790** 4.2 Additional Participant Information
Status: Approved

4.2 Additional Participant Information

1.0 **Describe the participants that will be included in this study. Outline ALL participants** *(i.e. if you are enrolling healthy controls as well):*

For saliva we have received samples that have already been collected from other studies by our colleagues: 1. A final examination stress study on college students and a longitudinal study associated with questionnaires of stress, anxiety and depression (male and female participants; Dr. Ritz). 2. Military personnel, pre-deployment training, during deployment in Afghanistan and post-deployment upon return to Canada (male and female participants; Dr. Cernak).3. Male and female participants in a study of stress and ulcerative colitis (Dr. Peerani) (serum and saliva samples).

In addition in our laboratory we will collect saliva from lab volunteers to create standards of saliva to be used in our immunoassays (<10 samples from males and females).

Dr. Stickland has collected samples from COPD subjects in Emergency Department and with follow-up at discharge from hospital and 14 days later. Additionally he has collected samples from stable COPD subjects.

2.0 * **Describe and justify the inclusion criteria for participants** *(e.g. age range, health status, gender, etc.):*

No restrictions on salivary gland tissue to be collected once it is marked to be discarded.

For serum and saliva studies are to be completed by our colleagues, and we are receiving the samples for secondary analyses of CABS1 proteins.

For the lab volunteers our inclusion criteria are healthy subjects willing to provide saliva.

3.0 **Describe and justify the exclusion criteria for participants:**

NA

4.0 **Participants**

4.1 How many participants do you hope to recruit *(including controls, if applicable?)*
1500
4.2 Of these, how many are controls, if applicable?
10
4.3 If this is a multi-site study, how many participants do you anticipate will be enrolled in the entire study?
1500

5.0 Justification for sample size:

Determined by colleagues for their studies; my analyses are secondary.

For our external standard of saliva; the sample size is based on an estimate of volume to be collected for our studies (estimate . 100 mL needed). This has been completed and aliquots are stored in our freezers.

ID: Pro00001790 **Pro00001790**
Status: Approved 4.4 Recruitment of Participants (non-Health)

4.4 Recruitment of Participants (non-Health)

1.0 Recruitment

1.1 How will you identify potential participants? Outline all of the means you will use to identify who may be eligible to be in the study*(i.e. response to advertising such as flyers, posters, ads in newspapers, websites, email, list serves, community organization referrals, etc.)*
Laboratory volunteers.

1.2 Once you have identified a list of potentially eligible participants, indicate how the potential participants' names will be passed on to the researchers AND how will the potential participants be approached about the research.
Word of mouth in the laboratory.

2.0 Pre-Existing Relationships

2.1 Will potential participants be recruited through pre-existing relationships with researchers*(e.g. Will an instructor recruit students from his classes, or a physician recruit patients from her practice? Other examples may be employees, acquaintances, own children or family members, etc.)*?
● **Yes** ○ No

2.2 If YES, identify the relationship between the researchers and participants that could compromise the freedom to decline*(e.g. clinician/patient, professor/student)*
The recruitment will be done by a MSc student, Mr Reyes-Serratos and participation will be on a volunteer basis.

2.3 How will you ensure that there is no undue pressure on the potential participants to agree to the study?
This will be stated very clearly and has been openly discussed. Those that have refused, have not been pressured.

3.0 **Will your study involve any of the following?** *(select all that apply)*
None of the above

4.5 Informed Consent Determination

1.0 **Describe who will provide informed consent for this study** *(i.e. the participant, parent of child participant, substitute decision maker, no one will give consent – requesting a waiver)*
Mr. Reyes-Serratos, the MSc student.

1.1 Waiver of Consent Requested
If you are asking for a waiver of participant consent, please justify the waiver or alteration and explain how the study meets all of the criteria for the waiver. Refer to Article 3.7 of TCPS2 **and provide justification for requesting a Waiver of Consent for ALL criteria (a-e)**
NA

1.2 Waiver of Consent in Individual Medical Emergency
If you are asking for a waiver or alteration of participant consent in individual medical emergencies, please justify the waiver or alteration and explain how the study meets ALL of the criteria outlined in Article 3.8 of TCPS2 (a-f)**.**

2.0 **How will consent be obtained/documented? Select all that apply**
Signed consent form

If you are not using a signed consent form, explain how the study information will be provided to the participant and how consent will be obtained/documented. Provide details for EACH of the options selected above:

3.0 **Will every participant have the capacity to give fully informed consent on his/her own behalf?**
● **Yes** ○ No

4.0 **What assistance will be provided to participants or those consenting on their behalf, who may require additional assistance?** *(e.g. non-English speakers, visually impaired, etc.)*

5.0 * **If at any time a PARTICIPANT wishes to withdraw from the study or from certain parts of the study, describe when and how this can be done.**
The volunteer can withdraw whenever they wish by a verbal indication.

6.0 **Describe the circumstances and limitations of DATA withdrawal from the study, including the last point at which participant DATA can be withdrawn** *(i.e. 2 weeks after transcription of interview notes)*

7.0 **Will this study involve any group(s) where non-participants are present? For example, classroom research might involve groups which include participants and non-participants.**

○ Yes ● **No**

ID: Pro00001790 **Pro00001790** 5.1 Data Collection
Status: Approved

5.1 Data Collection

1.0 * **Will the researcher or study team be able to identify any of the participants at <u>any stage</u> of the study?**

○ Yes ● **No**

2.0 **Primary/raw data collected will be** *(check all that apply)***:**

Anonymous - the information **NEVER** had identifiers associated with it (eg anonymous surveys) and risk of identification of individuals is low or very low

All personal identifying information removed (anonymized)

3.0 **If this study involves secondary use of data, list all original sources:**

Once we have analyzed the levels of CABS1 protein/fragments in the salivary samples we will discuss the data with our colleagues and decode the groups. Data on the groups is maintained in in Southern Methodist University, Dallas (Dr. Ritz), or by Dr. Ibolja Cernak (REIM study) or Drs. Peerani and Stickland U of A.

4.0 **In research where total anonymity and confidentiality is sought but cannot be guaranteed** *(eg. where participants talk in a group)* **how will confidentiality be achieved?**

NA; my lab will have no access to identification for secondary analyses.

ID: Pro00001790 **Pro00001790** 5.4 Data Storage, Retention, and Disposal
Status: Approved

5.4 Data Storage, Retention, and Disposal

1.0 * **Describe how research data will be stored, e.g. digital files, hard copies, audio recordings, other. Specify the physical location and how it will be secured to protect confidentiality and privacy.** *(For example, study documents must be kept in a locked filing cabinet and computer files are encrypted, etc. Write N/A if not applicable to your research)*

No subject identifiers are collected. Our data on CABS1 protein characteristics are stored in laboratory notebooks and in electronic files.

2.0 * **University policy requires that you keep your data for a minimum of 5 years following completion of the study but there is no limit on data retention. Specify any plans for future use of the data. <u>If the data will become part of a data repository or if this study involves the creation of a research database or registry for future research use, please provide details.</u>** <u>*(Write N/A if not applicable to your research)*</u>

Data will be used for publication and dissemination through common scientific channels. No plans exist for database or registries.

3.0

If you plan to destroy your data, describe when and how this will be done? Indicate your plans for the destruction of the identifiers at the earliest opportunity consistent with the conduct of the research and/or clinical needs:

ID: Pro00001790 **Pro00001790** Documentation
Status: Approved

Documentation

Add documents in this section according to the headers. Use Item 11.0 "Other Documents" for any material not specifically mentioned below.

Sample templates are available by clicking HERE.

1.0 Recruitment Materials:

Document Name	Version	Date	Description
There are no items to display			

2.0 Letter of Initial Contact:

Document Name	Version	Date	Description
There are no items to display			

3.0 Informed Consent / Information Document(s):

3.1 What is the reading level of the Informed Consent Form(s):
Grade 11

3.2 Informed Consent Form(s)/Information Document(s):

Document Name	Version	Date	Description
Information Consent Saliva.doc(0.01)	0.01	6/26/2018 1:46 PM	

4.0 Assent Forms:

Document Name	Version	Date	Description
Information Consent Saliva.doc(0.01)	0.01	6/26/2018 1:47 PM	

5.0 Questionnaires, Cover Letters, Surveys, Tests, Interview Scripts, etc.:

Document Name	Version	Date	Description
There are no items to display			

6.0 Protocol/Research Proposal:

Document Name	Version	Date	Description
CABS1 as a biomarker and potential therapeutic agent for stress(0.01)	0.01	4/21/2016 3:01 PM	
CABS1 and Stress April 21 2016(0.01)	0.01	4/21/2016 3:17 PM	
CABS1 2015 AJP(0.01)	0.01	4/21/2016 3:19 PM	
COPD CRIO Proposal_Final.pdf(0.01)	0.01	4/26/2021 1:40 PM	
Clinical Trials.gov protocol(0.01)	0.01	4/26/2021 1:41 PM	
COPD Stress Biomarker Protocol (secondary analysis) (0.01)	0.01	5/5/2021 2:15 PM	

7.0 **Investigator Brochures/Product Monographs:**

Document Name	Version	Date	Description
There are no items to display			

8.0 **Health Canada No Objection Letter** *(NOL)***:**

Document Name	Version	Date	Description
There are no items to display			

9.0 **Confidentiality Agreement:**

Document Name	Version	Date	Description
There are no items to display			

10.0 **Conflict of Interest:**

Document Name	Version	Date	Description
There are no items to display			

11.0 **Other Documents:**
For example, Study Budget, Course Outline, or other documents not mentioned above

Document Name	Version	Date	Description
Previous ethics application(0.01)	0.01	11/26/2012 3:05 PM	
Previous ethics approval(0.01)	0.01	11/26/2012 3:06 PM	
Ethics approval form, Dr. Ibolja Cernak, University of Alberta (Pro00035703)(0.01)	0.01	4/21/2016 3:58 PM	
Ethics approval letter, Dr. Thomas Ritz, Southern	0.01	4/21/2016 4:01 PM	

Document Name	Version	Date	Description
Methodist University(0.01)			

**ID:** Pro00001790	**Pro00001790**	Final Page
**Status:** Approved		

Final Page

You have reached the end of the ethics application.
Click ‘Continue’ or ‘Exit’ below.

To submit for ethics review, click “SUBMIT for REVIEW” on the left side of the screen.

NOTE: Only the Principal Investigator can submit an application in Pre-submission (ie: the first time it is submitted).

**ID:** Pro00001790	**Pro00001790**	Add/Edit Funding Info without Manual Entry
**Status:** Approved		

If you are trying to add a RES number in the ARISE application and you cannot find it on the drop down menu, please check the following:

1. *Check that the named investigators on your application match the people named on the RES account. RES numbers associated with anyone named as PI or Co-I on an ethics application will show up in the drop down box in Section 1.3 or in 6.0 of the Change Funding Activity. Please note that unless someone is named on the ethics application in either the PI or Co-I fields, their RES number(s) will NOT display in the drop down box of that application.*
2. *Check that the RES number you are trying to add has been activated by RSO (check unit name with RSO) and that 24 hours have elapsed since it was activated to allow time for system updates.*

If neither of the above items are the source of the issue, please contact reoffice@ualberta.ca.

Enter your Peoplesoft Project ID (aka RES#) to link this ethics application to the project record in PeopleSoft.

PeopleSoft Project ID:

Other Relevant Information:
Principal Investigator Dr. Marcelo Marcet 2020 - 2025-

**ID:** Pro00001790	**Pro00001790**	Add/Edit Funding Info without Manual Entry
**Status:** Approved		

If you are trying to add a RES number in the ARISE application and you cannot find it on the drop down menu, please check the following:

1. *Check that the named investigators on your application match the people named on the RES account. RES numbers associated with anyone named as PI or Co-I on an ethics application will show up in the drop down box in Section 1.3 or in 6.0 of the Change Funding Activity. Please note that unless someone is named on the ethics application in either the PI or Co-I fields, their RES number(s) will NOT display in the drop down box of that application.*
2. *Check that the RES number you are trying to add has been activated by RSO (check unit name with RSO) and that 24 hours have elapsed since it was activated to allow time for system updates.*

If neither of the above items are the source of the issue, please contact reoffice@ualberta.ca.

Enter your Peoplesoft Project ID (aka RES#) to link this ethics application to the project record in PeopleSoft.

PeopleSoft Project ID:
RES0058116

Other Relevant Information:

**ID:** Pro00001790	**Pro00001790**	
**Status:** Approved		Add/Edit Funding Info without Manual Entry

If you are trying to add a RES number in the ARISE application and you cannot find it on the drop down menu, please check the following:

1. *Check that the named investigators on your application match the people named on the RES account. RES numbers associated with anyone named as PI or Co-I on an ethics application will show up in the drop down box in Section 1.3 or in 6.0 of the Change Funding Activity. Please note that unless someone is named on the ethics application in either the PI or Co-I fields, their RES number(s) will NOT display in the drop down box of that application.*
2. *Check that the RES number you are trying to add has been activated by RSO (check unit name with RSO) and that 24 hours have elapsed since it was activated to allow time for system updates.*

If neither of the above items are the source of the issue, please contact reoffice@ualberta.ca.

Enter your Peoplesoft Project ID (aka RES#) to link this ethics application to the project record in PeopleSoft.

PeopleSoft Project ID:

Other Relevant Information:
2019-2022

**ID:** Pro00001790	**Pro00001790**	
**Status:** Approved		Add/Edit Funding Info without Manual Entry

If you are trying to add a RES number in the ARISE application and you cannot find it on the drop down menu, please check the following:

1. *Check that the named investigators on your application match the people named on the RES account. RES numbers associated with anyone named as PI or Co-I on an ethics application will show up in the drop down box in Section 1.3 or in 6.0 of the Change Funding Activity. Please note that unless someone is named on the ethics application in either the PI or Co-I fields, their RES number(s) will NOT display in the drop down box of that application.*
2. *Check that the RES number you are trying to add has been activated by RSO (check unit name with RSO) and that 24 hours have elapsed since it was activated to allow time for system updates.*

If neither of the above items are the source of the issue, please contact reoffice@ualberta.ca.

Enter your Peoplesoft Project ID (aka RES#) to link this ethics application to the project record in PeopleSoft.

PeopleSoft Project ID:

Other Relevant Information:
2005 - 2019

**ID:** Pro00001790	**Pro00001790**	
**Status:** Approved		Add/Edit Funding Info without Manual Entry

If you are trying to add a RES number in the ARISE application and you cannot find it on the drop down menu, please check the following:

1. *Check that the named investigators on your application match the people named on the RES account. RES numbers associated with anyone named as PI or Co-I on an ethics application will show up in the drop down box in Section 1.3 or in 6.0 of the Change Funding Activity. Please note that unless someone is*

named on the ethics application in either the PI or Co-I fields, their RES number(s) will NOT display in the drop down box of that application.

2. *Check that the RES number you are trying to add has been activated by RSO (check unit name with RSO) and that 24 hours have elapsed since it was activated to allow time for system updates.*

If neither of the above items are the source of the issue, please contact reoffice@ualberta.ca.

Enter your Peoplesoft Project ID (aka RES#) to link this ethics application to the project record in PeopleSoft.

PeopleSoft Project ID:

Other Relevant Information:
2016-2019

ID: Pro00001790 **Pro00001790**
Status: Approved Add/Edit Funding Info without Manual Entry

If you are trying to add a RES number in the ARISE application and you cannot find it on the drop down menu, please check the following:

1. *Check that the named investigators on your application match the people named on the RES account. RES numbers associated with anyone named as PI or Co-I on an ethics application will show up in the drop down box in Section 1.3 or in 6.0 of the Change Funding Activity. Please note that unless someone is named on the ethics application in either the PI or Co-I fields, their RES number(s) will NOT display in the drop down box of that application.*
2. *Check that the RES number you are trying to add has been activated by RSO (check unit name with RSO) and that 24 hours have elapsed since it was activated to allow time for system updates.*

If neither of the above items are the source of the issue, please contact reoffice@ualberta.ca.

Enter your Peoplesoft Project ID (aka RES#) to link this ethics application to the project record in PeopleSoft.

PeopleSoft Project ID:

Other Relevant Information:
This is a supplementary fund for Dr. Marcelo Marcet (NSERC)

Pro00001790 Reviewer Notes

No Reviewer notes to display.
