# Supplementary material for: Identification and characterization of calcium binding protein, spermatid-associated 1 (CABS1)# in selected human tissues and fluids
Source: PLoS One. 2024 May 16;19(5):e0301855. doi: 10.1371/journal.pone.0301855 (PMC11098423; doi:10.1371/journal.pone.0301855)
Supplement: S3 File — (PDF) [file pone.0301855.s008.pdf]

UNIVERSITY OF ALBERTA

ARISE
Alberta Research Information Services

Date: Tuesday, August 22, 2023 11:47:57 AM

Print Close

Table of Contents

Pro00112432

Packet Name: 2 - Smartform

- 1.1 Study Identification
- 1.2 Additional Approval
- 1.3 Funding Information
- 1.4 Conflict of Interest
- 1.5 Research Locations and Other Approvals
- 2.1 Study Objectives and Design
- 2.2 Research Methods and Procedures
- 2.21 Secondary Use of Human Biological Materials
- 3.1 Risk Assessment
- 3.2 Benefits Analysis
- 4.1 Participant Information
- 5.1 Data Collection
- 5.4 Data Storage, Retention, and Disposal
- Documentation
- Final Page
- Add/Edit Funding Info without Manual Entry (ID00060752)
- Add/Edit Funding Info without Manual Entry (ID00066275)

Packet Name: 3 - Reviewer Notes

- Reviewer Notes

1.1 Study Identification

*All questions marked by a red asterisk * are required fields. However, because the mandatory fields have been kept to a minimum, answering only the required fields may not be sufficient for the REB to review your application.*

Please answer all relevant questions that will reasonably help to describe your study or proposed research.

1.0 * **Short Study Title** *(restricted to 250 characters)*:
Distribution and cell-type localization of human CABS1

2.0 * **Complete Study Title** *(can be exactly the same as short title)*:
Immunohistochemical analysis of the tissue distribution and cell-type specific localization of human calcium-binding protein: spermatid-associated 1 (CABS1)

3.0 * **Select the appropriate Research Ethics Board (***Detailed descriptions are available at here***):**
HREB Biomedical

4.0 * **Is the proposed research:**
Funded (Grant, subgrant, contract, internal funds, donation or some other source of funding)

5.0 * **Name of local Principal Investigator:**
A. Dean Befus

6.0 * **Type of research/study:**
Faculty/Academic Staff

7.0 **Investigator's Supervisor***(required for applications from undergraduate students, graduate students, post-doctoral fellows and medical residents to REBs 1 & 2. HREB does not accept applications from student PIs)*:

8.0 **Study Coordinators or Research Assistants:** People listed here can edit this application and will receive all email notifications for the study:

Name	Employer
There are no items to display	

9.0 **Co-Investigators:**People listed here can edit this application and will receive email notifications *(Co-investigators who do not wish to receive email, should be added to the study email list team below instead of here).*

Name	Employer
Marcelo Marcet-Palacios	MH Medicine
Lakshmi Puttagunta	MH Laboratory Med & Pathology

10.0 **Primary Admin Contact:**
A. Dean Befus

11.0 **Study Team:***(Co-investigators, supervising team, other study team members) - People listed here cannot view or edit this application and do not receive email notifications.*

Last Name	First Name	Organization	Role/Area of Responsibility	Phone	Email
Ramielle Santos	Joy	University of Alberta	MSc Student working on CABS1	780-908-4442	joyramie@ualberta.ca
Reyes-Serratos	Eduardo	University of Alberta	PhD student working on detection and molecular processing of CABS1	780-200-2382	reyesser@ualberta.ca
Canil	Sarah	Alberta Precision Labs	Co-investigator		sarah.canil@albertaprecisionlabs.ca

ID: Pro00112432 **Pro00112432** 1.2 Additional Approval
Status: Approved

1.2 Additional Approval

1.0 * **Departmental Review:** ***Please note only ONE Department Review is required. Please ensure that this section reflects only the PRIMARY Department of the study PI.***

MH Medicine

2.0 **Internal Review** *(If the Principal Investigator is in the Department of Medicine complete the Department of Medicine Request for Internal Approval form and upload it to the "Documentation" section of this application under item 11.0 "Other Documents". Note that all fields in the form are required. The form is available at here)***:**

Medicine

ID: Pro00112432 **Pro00112432** 1.3 Funding Information
Status: Approved

1.3 Study Funding Information

1.0 * **Type of Funding:**
Grant (external)
Internal Funds (eg. Start-up funds, TLEF, Operational, etc)

2.0 * **Indicate which office administers your award.** *(It is the PI's responsibility to provide ethics approval notification to any office other than the ones listed below)*
University of Alberta - Research Services Office (RSO)

To connect your ethics application with your funding: provide all identifying information about the study funding – multiple rows allowed. For Project ID, enter a Funding ID provided by RSO/PeopleSoft Project ID*(for example, RES0005638, G018903401, C19900137, etc).***Enter the corresponding title for each Project ID.**

	Project ID	Title	Grant Status	Sponsor	Project Start Date	Project End Date	Purpose	Other Information
View	RES0048542	Differential expression and processing of CABS1 across tissues and fluids and its location specific functions.	Awarded	Discov Grt Individual	4/1/2020	3/31/2025	Grant	
View	RES0058116	Gen Res - Befus,A. Dean	Submitted	General Research Project (PI)	3/1/2022	3/31/2025	General Research	

3.0 * **Funding Source**

3.1 Select all sources of funding from the list below:
NSERC - Natural Sciences And Engineering Research Council NSERC

3.2 If your source of funding is not available in the list above, click "Add" below and write the Sponsor/Agency name(s) in the free text box that pops up.*(Nore: You may reflect multiple sources of funding by continuing to click "Add" to add each additional source of funding).*
Faculty of Medicine and Dentistry - Transition to Retirement funding to Dr. Dean Befus (TRPDB)
GENRES Account U of A

4.0 * **Indicate if this research sponsored or monitored by any of the following:**
Not applicable

The researcher is responsible for ensuring that the study complies with the applicable US regulations. The REB must also comply with US Regulations.

ID: Pro00112432 **Pro00112432** 1.4 Conflict of Interest
Status: Approved

1.4 Conflict of Interest

1.0 * **Are any of the investigators or their immediate family receiving any personal remuneration (including investigator payments and recruitment incentives but excluding trainee remuneration or graduate student stipends) from the funding of this study that is not accounted for in the study budget?**

○ Yes ● **No**

2.0 * **Do any of investigators or their immediate family have any proprietary interests in the product under study or the outcome of the research including patents, trademarks, copyrights, and licensing agreements?**

● **Yes** ○ No

3.0 * **Is there any compensation for this study that is affected by the study outcome?**

○ Yes ● **No**

4.0 * **Do any of the investigators or their immediate family have equity interest in the sponsoring company? (This does not include Mutual Funds)**

○ Yes ● **No**

5.0 * **Do any of the investigators or their immediate family receive payments of other sorts, from this sponsor (i.e. grants, compensation in the form of equipment or supplies, retainers for ongoing consultation and honoraria)?**

○ Yes ● **No**

6.0 * **Are any of the investigators or their immediate family, members of the sponsor's Board of Directors, Scientific Advisory Panel or comparable body?**

○ Yes ● **No**

7.0 * **Do you have any other relationship, financial or non-financial, that, if not disclosed, could be construed as a conflict of interest?**

○ Yes ● **No**

Please explain if the answer to any of the above questions is Yes:
Dr. Befus is the co-holder, with the University of Alberta, of a patent on CABS1. In addition, there is a licensing agreement with GB Diagnostics for the use of this patent as a biomarker of stress. The current proposal is not focused on stress or any specific disease, but is a descriptive study of the distribution of CABS1 in selected human tissues.

Important

If you answered YES to any of the questions above, you may be asked for more information.

1.5 Research Locations and Other Approvals

1.0 *** List the locations of the proposed research, including recruitment activities. Provide name of institution, facility or organization, town, or province as applicable**

Dr. Lakshmi Puttagunta, Pathologist, will collect up to five human tissue samples (salivary glands, testes, lung, gastrointestinal tract, and adrenal glands) that had been acquired at surgery and that would otherwise be discarded. Samples will be acquired from surgeries performed at Alberta Health Services facilities in the Edmonton Zone and stored in Alberta Precision Laboratories or Dynalife Laboratories. They will be used to assess the tissue and cell type distribution of CABS1 in these human tissues.

This new study proposal is closely linked with our study Pro00001790, entitled Anti-inflammatory proteins and biomarkers of stress.

2.0 *** Indicate if the study will use or access facilities, programmes, resources, staff, students, specimens, patients or their records, at any of the sites affiliated with the following** *(select all that apply):*

Alberta Health Services Institutions and Facilities
Covenant Health Institutions and Facilities
Capital Care Institutions and Facilities

List all health care research sites/locations:

Surgical specimens of human testes, salivary glands, lung, gastrointestinal tract and adrenal glands that would otherwise be discarded will be collected through AHS resources at Edmonton Zone facilities, particularly the UofA hospital and potentially the Royal Alexandra Hospital, Edmonton.

3.0

Multi-Institution Review

*** 3.1 Has this study already received approval from another REB?**

○ **Yes** ● **No**

4.0 **If this application is closely linked to research previously approved by one of the University of Alberta REBs or has already received ethics approval from an external ethics review board***(s)***, provide the study number, REB name or other identifying information. Attach any external REB application and approval letter in the Documentation Section – Other Documents.**

MS22_Pro00001790; Anti-inflammatory proteins and biomarkers of stress.

2.1 Study Objectives and Design

1.0 *** Provide a lay summary of your proposed research which would be understandable to general public**

In studies in an experimental model of inflammatory disease in rats, we identified a pathway regulated by the nervous system (a mind-body pathway?) that can inhibit inflammatory responses. Interestingly, the source of the anti-inflammatory activity is in the salivary glands.

An important research priority was whether or not this anti-inflammatory pathway exists in humans and if human salivary glands express the same gene or, at least a closely related gene, and whether or not it has anti-inflammatory activity. We discovered that the human does not have the same gene as in the rat, but we have identified a related gene (CABS1) and established that a fragment from the protein can reduce inflammation. Moreover, to study if this pathway is under the control of the nervous system, we tested if stress in humans would change the levels of this protein in saliva. We discovered that stress enhances the levels of the protein. Indeed some smaller forms of the protein appear to identify individuals who are resistant to some of the effects of stress; they are resilient.

We now propose to conduct an immunohistochemical analysis of the tissue and cell-type distribution of CABS1 in normal salivary glands, and in other tissues where there is some evidence of CABS1 presence, namely, testes, lung, gastrointestinal tract and adrenal glands; ie, exactly where is this protein found in different cells and anatomical compartments in these tissues. This work will be done with in partnership with our long-term colleague Dr. Lakshmi Puttagunta, U of A, a highly skilled anatomist and pathologist experienced with these various tissues.

We will access archived (otherwise to be discarded) samples of these tissues collected from surgical specimens (3 to 5 independent samples of each tissue) from Edmonton Zone clinical laboratory facilities. We will work with Dr. Sarah Canil, Alberta Precision Laboratories, to optimize the methods to identify CABS1 in these tissues using staining procedures with antibodies to CABS1 that we have produced. Once the slides from the tissues are stained, we will review them carefully using microscopy and determine the distribution, abundance and cell-type localization of CABS1. Depending on the results of our work, we may decide to seek approval for additional studies on the distribution and abundance of CABS1 in diseased tissues. We anticipate publishing this descriptive work in an appropriate, internationally recognized peer-reviewed journal.

Future work could involve determining whether psychosocial or other interventions might enhance this mind-body pathway to reduce stress, modulate inflammatory responses and alter their effects on human health.

2.0 * **Provide a full description of your research proposal outlining the following:**

- **Purpose**
- **Hypothesis**
- **Justification**
- **Objectives**
- **Research Method/Procedures**
- **Plan for Data Analysis**

Purpose

The purpose of this study is to identify the distribution, precise anatomical location and specific cell-type localization of CABS1 in human tissues.

Hypothesis

CABS1 is more widely distributed in human tissues than previous literature has identified and is localized in epithelial cell compartments, not only in urogenital tract (testes), but also in lung, gastrointestinal tract, including salivary glands, and in adrenal glands.

Justification

The research in our laboratory is focused on the analysis of CABS1 protein and its putative post-translationally processed molecular forms, both as biomarkers of stress and potentially as anti-inflammatory mediators. We have established that CABS1 is expressed in human salivary glands and lungs, as well as testes (St. Laurent et al., 2015, attached) and that a peptide sequence near its carboxyl terminus has anti-inflammatory activity. Moreover, in three independent studies (baseline, acute stress and chronic stress), CABS1 levels in saliva can be modified during stress (Ritz et al., 2017; attached).

As a result of these discoveries, we have an approved patent with the University of Alberta, and are working towards further validation of our discoveries and extension of the results and their implications in human health and disease. This work was all covered under the ethically approved study, Pro00001790.

Apart from studies of CABS1 in rodent and bovine testes, their have been few studies to determine the tissue distribution of CABS1 protein, and no published studies in human tissues apart from our work largely on salivary glands (see above). In a previous (unpublished) collaboration with colleagues in Case Western Reserve University, Cleveland, Ohio, we have initial observations of the localization of CABS1 in the acinar and ductal epithelium of human submandibular glands. We now wish to do a much more extensive investigation of the tissue distribution, precise anatomical compartments and cell-type specific localization of CABS1 in tissues where mRNA or proteomic studies have suggested the presence of CABS1. These tissues include, male and female urogenital tract, lung, salivary glands, gastrointestinal tract and adrenal glands (association with stress).

An more complete understanding of the tissue and cell-type distribution of CABS1 in humans will provide a fundamental basis for future targeted studies of its regulation and functions.

Objectives

1. Receive ethics approval

2. Acquire samples of clinically-necessary surgical specimens of male and female urogenital tract, salivary glands, gastrointestinal tract, lungs and adrenal glands. The samples will be identified by Dr. Puttagunta from archived (otherwise to be discarded) specimens in clinical laboratory facilities of the Edmonton Zone.

3. In collaboration with Dr. Sarah Canil, Alberta Precision laboratories, optimize the immunohistochemical protocol to stain CABS1 in tissue sections on microscope slides. Monoclonal antibodies to CABS1 from our laboratories will be used for the immunostaining.

4. Stain slides from 3 to 5 independent samples of each tissue.

5. Systematically determine if CABS1 is expressed in each of the tissues and identify the anatomical locations and cell-type specific localization of CABS1.

6. Disseminate the new knowledge that we gain on CABS1 through presentations at scientific meetings and in peer-reviewed publication(s) in internationally respected scientific journals.

Research Method/Procedures

The description of our objectives (above) outlines our core research approaches and methodology.

We have produced three monoclonal antibodies to different regions of CABS1 protein that will be optimized for the staining protocols and used to identify CABS1. We will cut representative sections of each tissue and its core anatomical compartments and using light microscopy will describe the localization of CABS1. Given that we have evidence for multiple forms of CABS1, we will determine if each of the antibodies produces similar results, or if some forms may have a different tissue/cell-type distribution that others.

Plan for data analysis

This is a descriptive anatomical study of the distribution of human CABS1. We will use multiple slides from each tissue sample and 3 to 5 independent samples of each tissue. We do not anticipate the need for any statistical analyses to describe our observations.

3.0 **Describe procedures, treatment, or activities that are above or in addition to standard practices in this study area** *(eg. extra medical or health-related procedures, curriculum enhancements, extra follow-up, etc)***:**
Not applicable.

4.0 **If the proposed research is above minimal risk and is not funded via a competitive peer review grant or industry-sponsored clinical trial, the REB will require evidence of scientific review. Provide information about the review process and its results if appropriate.**
The study has minimal risk and is funded in part by NSERC (peer reviewed).

5.0 **For clinical trials, describe any sub-studies associated with this Protocol.**
Not applicable.

ID: Pro00112432 **Pro00112432**
Status: Approved 2.2 Research Methods and Procedures

2.2 Research Methods and Procedures

Some research methods prompt specific ethical issues. The methods listed below have additional questions associated with them in this application. If your research does not involve any of the methods listed below, ensure that your proposed research is adequately described in Section 2.1: Study Objectives and Design or attach documents in the Documentation Section if necessary.

1.0 * **This study will involve the following***(select all that apply)*
Secondary Use of Human Biological Materials - *See NOTE 2 below*

NOTE 1: Select this if you are directly collecting health information as part of your protocol OR will be conducting a chart/record review/reviewing health data secondarily. This includes anonymized or identifiable health information.

NOTE 2: Select this option if this research ONLY involves analysis of blood/tissue/specimens originally collected for another purpose but now being used to answer your research question. If you are enrolling people into the study to prospectively collect specimens to analyze you SHOULD NOT select this box.

NOTE 3: This section is intended to reflect the secondary use of non-health data. Do NOT select this if you are using data that originally came from health sources, i.e., anonymized administrative data.

2.21 Secondary Use of Human Biological Materials

1.0 Outline where will you be getting the human biological materials from?
For the immunohistochemical studies of CABS1 distribution and cell-type localization, Dr. Lakshmi Puttagunta, a pathologist will select tissue blocks of urogenital tract, submandibular gland, lung, gastrointestinal tract and adrenal glands from archived samples in Edmonton zone clinical laboratory facilities. Samples of the necessary tissues will be found by searching the laboratory medicine information system database for archived tissues.

2.0 How/under what authority were these human biological materials originally collected?*(i.e. clinical specimens now being used for research, collected under a previous research protocol)*
These surgical cases will have been reported and completed for clinical diagnostic purposes. This tissue is considered discarded tissue as it is currently stored. There will be no further procedures on a patient to acquire the tissue needed for this study.
At the time of the original surgery performed, each patient would have signed a consent to proceed with the surgery and to retain the tissue for research and teaching purposes. Given that the material being used for this study is entirely archival tissues, there is no risk to the patient's safety or well being.

3.0 If specimens were originally collected under a research protocol, please outline how the proposed use of the samples is consistent with the parameters or restrictions of use described at the time of initial collection*(i.e. consent for future use was outlined in original consent form or ethics approval documentation)*
Not applicable.

4.0 Are the human biological materials you will be receiving/using:
Non-identifiable (i.e. you will not receive any identifiable health information linked to the specimens, nor would you ever be able to identify who the specimen came from)

3.1 Risk Assessment

1.0 * **Provide your assessment of the risks that may be associated with this research:**
Minimal Risk - research in which the probability and magnitude of possible harms implied by participation is no greater than those encountered by participants in those aspects of their everyday life that relate to the research (TCPS2)

2.0 * **Select all that might apply:**

**Description of Possible Physical Risks and Discomforts**	
No	Participants might feel physical fatigue, e.g. sleep deprivation
No	Participants might feel physical stress, e.g. cardiovascular stress tests
No	Participants might sustain injury, infection, and intervention side-effects or complications
No	The physical risks will be greater than those encountered by the participants in everyday life

**Possible Psychological, Emotional, Social and Other Risks and Discomforts**	
No	Participants might feel psychologically or emotionally stressed, demeaned, embarrassed, worried, anxious, scared or distressed, e.g. description of painful or traumatic events
No	Participants might feel psychological or mental fatigue, e.g intense concentration required
No	Participants might experience cultural or social risk, e.g. loss of privacy or status or damage to reputation
No	Participants might be exposed to economic or legal risk, for instance non-anonymized workplace surveys
No	The risks will be greater than those encountered by the participants in everyday life

3.0 * **Provide details of all the risks and discomforts associated with the research for which you indicated YES or POSSIBLY above.**
Not applicable.

4.0 * **Describe how you will manage and minimize risks and discomforts, as well as mitigate harm:**
Not applicable. This study is of archived surgical samples (otherwise to be discarded).

5.0 **Is there a possibility that your research procedures will lead to unexpected findings, adverse reactions, or similar results that may require follow-up** *(i.e. individuals disclose that they are upset or distressed during an interview/questionnaire, unanticipated findings on MRI, etc.)***?**
○ Yes ● **No**

6.0 **If you are using any tests in this study diagnostically, indicate the member(s) of the study team who will administer the measures/instruments:**

Test Name	Test Administrator	Organization	Administrator's Qualification
There are no items to display			

7.0 **If any research related procedures/tests could be interpreted diagnostically, will these be reported back to the participants and if so, how and by whom?**
not applicable

ID: Pro00112432 **Pro00112432** 3.2 Benefits Analysis
Status: Approved

3.2 Benefits Analysis

1.0 * **Describe any potential benefits of the proposed research to the participants. If there are no benefits, state this explicitly:**
There are no anticipated benefits to the subjects.

2.0 * **Describe the scientific and/or scholarly benefits of the proposed research:**
The research will extend knowledge of the normal distribution of CABS1 in various tissues, their anatomical compartments and in specific cell types. Such knowledge will be foundational for future studies of the regulation of CABS1 expression and its functional significance in health and disease.

3.0 **If this research involves risk to participants explain how the benefits outweigh the risks.**
not applicable.

ID: Pro00112432 **Pro00112432** 4.1 Participant Information
Status: Approved

4.1 Participant Information

1.0 * **Will you be recruiting human participants** *(i.e. enrolling people into the study, sending people online surveys to complete)***?**
○ Yes ● **No**

ID: Pro00112432 **Pro00112432** 5.1 Data Collection
Status: Approved

5.1 Data Collection

1.0 * **Will the researcher or study team be able to identify any of the participants at any stage of the study?**
○ Yes ● **No**

2.0 **Primary/raw data collected will be** *(check all that apply)***:**

All personal identifying information removed (anonymized)

3.0 **If this study involves secondary use of data, list all original sources:**
Not applicable.

4.0 **In research where total anonymity and confidentiality is sought but cannot be guaranteed** *(eg. where participants talk in a group)* **how will confidentiality be achieved?**
not applicable.

5.4 Data Storage, Retention, and Disposal

1.0 * **Describe how research data will be stored, e.g. digital files, hard copies, audio recordings, other. Specify the physical location and how it will be secured to protect confidentiality and privacy.** *(For example, study documents must be kept in a locked filing cabinet and computer files are encrypted, etc. Write N/A if not applicable to your research)*
The research data will be a descriptive anatomical localization of CABS1 in tissues and cell types in males and females. Lab note books and Word files will contain the data. In addition, photomicrographs will be taken of representative distribution of CABS1 in each tissue and in specific cell types in anatomical compartments of the tissues. The data files will be stored in the offices of Drs. Lakshmi Puttagunta and Dean Befus.

2.0 * **University policy requires that you keep your data for a minimum of 5 years following completion of the study but there is no limit on data retention. Specify any plans for future use of the data. <u>If the data will become part of a data repository or if this study involves the creation of a research database or registry for future research use, please provide details.</u>** <u>*(Write N/A if not applicable to your research)*</u>
We do not anticipate use of the data in the future, beyond what will be published in our descriptive study of the normal distribution of CABS1.

3.0

If you plan to destroy your data, describe when and how this will be done? Indicate your plans for the destruction of the identifiers at the earliest opportunity consistent with the conduct of the research and/or clinical needs:
We do not have specific plans to destroy the data, other than what would occur once the entire CABS1 research program is terminated. This will be influenced by long-term funding or future retirements.

Documentation

Add documents in this section according to the headers. Use Item 11.0 "Other Documents" for any material not specifically mentioned below.

Sample templates are available by clicking HERE.

1.0 Recruitment Materials:

Document Name	Version	Date	Description
There are no items to display			

2.0 Letter of Initial Contact:

Document Name	Version	Date	Description
There are no items to display			

3.0 Informed Consent / Information Document(s):

3.1 What is the reading level of the Informed Consent Form(s):

3.2 Informed Consent Form(s)/Information Document(s):

Document Name	Version	Date	Description
General Surgery Consent form (0.01)	0.01	7/14/2021 2:43 PM	

4.0 Assent Forms:

Document Name	Version	Date	Description
There are no items to display			

5.0 Questionnaires, Cover Letters, Surveys, Tests, Interview Scripts, etc.:

Document Name	Version	Date	Description
There are no items to display			

6.0 Protocol/Research Proposal:

Document Name	Version	Date	Description
Immunohistochemical Protocol CABS1(0.01)	0.01	7/14/2021 2:49 PM	

7.0 Investigator Brochures/Product Monographs:

Document Name	Version	Date	Description
There are no items to display			

8.0 Health Canada No Objection Letter *(NOL)*:

Document Name	Version	Date	Description
There are no items to display			

9.0 Confidentiality Agreement:

Document Name	Version	Date	Description
There are no items to display			

10.0 Conflict of Interest:

Document Name	Version	Date	Description
There are no items to display			

11.0 Other Documents:
For example, Study Budget, Course Outline, or other documents not mentioned above

Document Name	Version	Date	Description
CABS1 and Stress 2017.pdf(0.01)	0.01	7/7/2021 3:09 PM	
CABS1 2015 AJP.pdf(0.01)	0.01	7/7/2021 3:10 PM	
DoM Ethics Proposal internal approval07072021.pdf(0.01)	0.01	7/7/2021 3:18 PM	
Alberta Precision Laboratories Budget (0.01)	0.01	7/14/2021 2:41 PM	

ID: Pro00112432 **Pro00112432** Final Page
Status: Approved

Final Page

You have reached the end of the ethics application.
Click ‘Continue’ or ‘Exit’ below.

To submit for ethics review, click “SUBMIT for REVIEW” on the left side of the screen.

NOTE: Only the Principal Investigator can submit an application in Pre-submission (ie: the first time it is submitted).

ID: Pro00112432 **Pro00112432** Add/Edit Funding Info without Manual Entry
Status: Approved

If you are trying to add a RES number in the ARISE application and you cannot find it on the drop down menu, please check the following:

1. *Check that the named investigators on your application match the people named on the RES account. RES numbers associated with anyone named as PI or Co-I on an ethics application will show up in the drop down box in Section 1.3 or in 6.0 of the Change Funding Activity. Please note that unless someone is named on the ethics application in either the PI or Co-I fields, their RES number(s) will NOT display in the drop down box of that application.*
2. *Check that the RES number you are trying to add has been activated by RSO (check unit name with RSO) and that 24 hours have elapsed since it was activated to allow time for system updates.*

If neither of the above items are the source of the issue, please contact reoffice@ualberta.ca.

Enter your Peoplesoft Project ID (aka RES#) to link this ethics application to the project record in PeopleSoft.

PeopleSoft Project ID:
RES0048542

Other Relevant Information:

ID: Pro00112432 **Pro00112432**
Status: Approved Add/Edit Funding Info without Manual Entry

If you are trying to add a RES number in the ARISE application and you cannot find it on the drop down menu, please check the following:

1. *Check that the named investigators on your application match the people named on the RES account. RES numbers associated with anyone named as PI or Co-I on an ethics application will show up in the drop down box in Section 1.3 or in 6.0 of the Change Funding Activity. Please note that unless someone is named on the ethics application in either the PI or Co-I fields, their RES number(s) will NOT display in the drop down box of that application.*
2. *Check that the RES number you are trying to add has been activated by RSO (check unit name with RSO) and that 24 hours have elapsed since it was activated to allow time for system updates.*

If neither of the above items are the source of the issue, please contact reoffice@ualberta.ca.

Enter your Peoplesoft Project ID (aka RES#) to link this ethics application to the project record in PeopleSoft.

PeopleSoft Project ID:
RES0058116

Other Relevant Information:

Pro00112432 Reviewer Notes

No Reviewer notes to display.
