# Supplementary material for: Identification and characterization of calcium binding protein, spermatid-associated 1 (CABS1)# in selected human tissues and fluids
Source: PLoS One. 2024 May 16;19(5):e0301855. doi: 10.1371/journal.pone.0301855 (PMC11098423; doi:10.1371/journal.pone.0301855)
Supplement: S4 File — (PDF) [file pone.0301855.s009.pdf]

UNIVERSITY OF ALBERTA

RESEARCH ETHICS
Health Research Ethic

2-01 North Power Pl
11312 - 8
Edmonton, Alberta, Canada
Tel: 780
www.u

Re-Approval Form

Date:	May 3, 2023
Renewal ID:	Pro00001790_REN16
Principal Investigator:	A. Dean Befus
Study ID:	Pro00001790
Study Title:	Anti-inflammatory proteins and biomarkers of stress
Approval Expiry Date:	May 1, 2024
Protocol Number:	Pro00038838 (Dr. Stickland)
Sponsor/Funding Agency:	Network of Centres of Excellence NCE<br>NSERC - Natural Sciences And Engineering Research Council NSERC<br>GBD Inc. RES0044356

The Health Research Ethics Board - Biomedical Panel has reviewed the renewal request and file for this project and found it to be acceptable within the limitations of human experimentation.

The re-approval for the study as presented is valid for another year. It may be extended following completion of the annual renewal request. Beginning 30 days prior to expiration, you will receive notices that the study is about to expire. Once the study has expired you will have to resubmit. Any proposed changes to the study must be submitted to the HREB for approval prior to implementation.

All study-related documents should be retained so as to be available to the HREB on request. They should be kept for the duration of the project and for at least five years following study completion. In the case of clinical trials approved under Division 5 of the Food and Drug regulations of Health Canada, study records must be retained for 15 years.

The membership of the Health Research Ethics Board - Biomedical Panel complies with the membership requirements for research ethics boards as defined in Division 5 of the Food and Drug Regulations and the Tri Council Policy Statement. The HREB - Biomedical Panel carries out its functions in a manner consistent with Good Clinical Practices.

Approval by the REB does not constitute authorization to initiate the conduct of this research. The Principal Investigator is responsible for ensuring required approvals from other involved organizations (e.g., Alberta Health Services, Covenant Health, community organizations, school boards) are obtained, before the research begins.

Sincerely,

Emily Nolan
REB Specialist
On behalf of
S.K.M. Kimber, MD, FRCPC
Chair, HREB Biomedical

Note: This correspondence includes an electronic signature (validation and approval via an online system).

Alberta Health Services

Covenant Health
