# Supplementary material for: Identification and characterization of calcium binding protein, spermatid-associated 1 (CABS1)# in selected human tissues and fluids
Source: PLoS One. 2024 May 16;19(5):e0301855. doi: 10.1371/journal.pone.0301855 (PMC11098423; doi:10.1371/journal.pone.0301855)
Supplement: S6 File — (DOCX) [file pone.0301855.s011.docx]

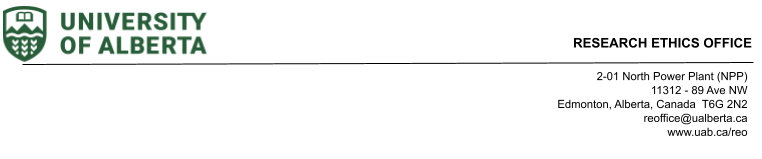
September 11, 2023

Sruthi Menon

Editorial Office

PLOS ONE

Re: PONE-D-23-19407

Identification and Characterization of Calcium Binding Protein, Permatid Associated 1 (CABS1) in Selected Human Tissues and Fluids Co-Investigators: Drs. A. Dean Befus and Marcelo Marcet-Palacios

Dear Ms. Menon,

I am writing in regards to the human ethics approval of the above submission to PLOS ONE. This study is covered by two protocols that received approval from the University of Alberta Health Research Ethic Board. Pro00001790, Anti-inflammatory Proteins and Biomarkers of Stress, is a longstanding protocol that was originally approved for the secondary analysis of human biological materials normally discarded, as well as for human serum and saliva. The second protocol, Pro00112432 is entitled: Immunohistochemical analysis of the tissue distribution and cell-type specific localization of human calcium binding protein, spermatid-associated protein 1.

The Research Ethics Board (REB) determined that specific research consent for the secondary use of surgical tissues materials from both of these studies could be waived outside of the broad consent provided by the patient at the time of their procedure. The initial study, Pro0001790 was reviewed and approved in May, 2007, and annual reapprovals have been maintained since then. The study is currently open with an expiration date of May 1, 2024. Pro00112432 was initially approved July 30, 2021, has been renewed twice and is currently active until the next renewal deadline, June 12, 2024.

The study investigators will provide information of these Human Ethics Protocols in their manuscript. I am happy to provide any further details as required to demonstrate our rigorous ethical considerations of the aforementioned studies.

Sincerely,


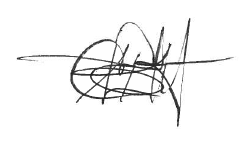


Charmaine Kabatoff

Senior Officer, REB,

Research Ethics, Research Integrity Support
