# Supplementary material for: Identification and characterization of calcium binding protein, spermatid-associated 1 (CABS1)# in selected human tissues and fluids
Source: PLoS One. 2024 May 16;19(5):e0301855. doi: 10.1371/journal.pone.0301855 (PMC11098423; doi:10.1371/journal.pone.0301855)
Supplement: S1 File — (PDF) [file pone.0301855.s006.pdf]

UNIVERSITY OF ALBERTA

August 17, 2023

Dr. Melanie Española
Straive Editorial Assistant
PLOS ONE

Subject: Animal ethics review of antibody production by commercial suppliers – Review of Genscript Animal Study. Principal Investigator: Dr. Dean Befus
Genscript Biotech IACUC# ANT17-003
Genscript Biotech IACUC#ANT14-001

Dear Dr. Española,

I am writing to inform you of the University of Alberta's decision regarding the research activities conducted for Dr. Befus at GenScript Biotech Corporation. Our institution has carefully evaluated the nature of the research carried out by Dr. Befus and its alignment with our guidelines and regulations pertaining to animal research.

The University of Alberta places great emphasis on maintaining the highest standards of ethical and responsible animal research. It is with this commitment in mind that we have thoroughly reviewed the collaboration between Dr. Befus and Genscript, and the latter's involvement in antibody production. Our assessment has led us to conclude that the protocols and practices followed by Genscript are in accordance with our Sustainable Purchasing guideline and code, which encompasses activities of this nature.

Based on our evaluation, the University of Alberta does not require a separate Animal Care and Use Committee (ACUC) review of antibody production conducted by commercial suppliers, including Genscript. Our thorough assessment has indicated that Genscript's procedures align with the guidelines set forth by our institution, and they have demonstrated the possession of the required certifications and accreditations necessary for carrying out this work within their jurisdiction. These certifications include the Office of Laboratory Animal Welfare (OLAW) and the Association for Assessment and Accreditation of Laboratory Animal Care (AAALAC) accreditations, along with approval from the Institutional Animal Care and Use Committee (IACUC).

Vice-President (Research and Innovation)
Research Ethics Office – Research Integrity Support

2-01 North Power Plant (NPP)
11312 - 89 Ave NW
Edmonton, AB, Canada T6G 2N2

reoffice@ualberta.ca
T 780.492.0459
uab.ca/reo

UNIVERSITY OF ALBERTA

We want to assure you that the University of Alberta takes its responsibilities in animal research seriously and only acknowledges protocols that adhere to the highest ethical and regulatory standards. Therefore, we have accepted Genscript's protocol as an equivalent and approved protocol for the specified research activities.

It is important to note that our institution does not engage in retrospective review or approval of Animal Use Protocols. However, in the case of Genscript, their protocols have been found to fall within the parameters where the University is not obligated, as per the Canadian Council on Animal Care (CCAC) regulations, to conduct a separate review for this specific type of activity.

If you have any further inquiries or require additional information regarding our evaluation of Genscript's protocols, please do not hesitate to reach out to us. We are committed to transparency and accountability in our animal research practices and are more than willing to address any concerns you may have.

Sincerely,

DVM.

Dr. Craig Wilkinson
University Veterinarian

cc: Pamela Raposo – Senior Officer Animal Care and Use
